# Association of the Home Health Value-Based Purchasing Model With Quality, Utilization, and Medicare Payments After the First 5 Years

**DOI:** 10.1001/jamahealthforum.2022.2723

**Published:** 2022-09-23

**Authors:** Alyssa Pozniak, Eric Lammers, Purna Mukhopadhyay, Chad Cogan, Zhechen Ding, Rashmi Goyat, Katherine Hanslits, Nan Ji, Yan Jin, Kaitlyn Repeck, Jillian Schrager, Eric Young, Marc Turenne

**Affiliations:** 1 Arbor Research Collaborative for Health, Ann Arbor, Michigan

## Abstract

**Importance:**

The original Home Health Value-Based Purchasing (HHVBP) model provided financial incentives to home health agencies for quality improvement in 9 randomly selected US states.

**Objective:**

To evaluate quality, utilization, and Medicare payments for home health patients in HHVBP states compared with those in comparison states.

**Design, Setting, and Participants:**

This cohort study was conducted in 2021 with secondary data from January 2013 to December 2020. A difference-in-differences design and multivariate linear regression were used to compare outcomes for Medicare and Medicaid beneficiaries who received home health care in HHVBP states with those in 41 comparison states during 3 years of preintervention (2013-2015) and the subsequent 5 years (2016-2020).

**Exposures:**

Home health care provided by a home health agency in HHVBP states and comparison states.

**Main Outcomes and Measures:**

Utilization (unplanned hospitalizations, emergency department visits, skilled nursing facility [SNF] visits) for Medicare beneficiaries within 60 days of beginning home health, Medicare payments during and 37 days after home health episodes, and quality of care (functional status, patient experience) during home health episodes.

**Results:**

Among 34 058 796 home health episodes (16 584 870 beneficiaries; mean [SD] age of 76.6 [11.7] years; 60.5% female; 11.2% Black non-Hispanic; 79.5% White non-Hispanic) from January 2016 to December 2020, 22.6% were in HHVBP states and 77.4% were in non-HHVBP states. For the HHVBP and non-HHVBP groups, 60.4% and 61.0% of episodes were provided to female patients; 10.0% and 13.6% were provided to Black non-Hispanic patients, and 82.4% and 75.2% were provided to White non-Hispanic patients, respectively. Unplanned hospitalizations decreased by 0.15 percentage points (95% CI, –0.30 to –0.01) more in HHVBP states, a 1.0% decline compared with 15.7% at baseline. The use of SNFs decreased by 0.34 percentage points (95% CI, –0.40 to –0.27) more in HHVBP states, a 6.9% decline compared with the 4.9% baseline average. There was an association between HHVBP and a reduction in average Medicare payments per day of $2.17 (95% CI, –$3.67 to –$0.68) in HHVBP states, primarily associated with reduced inpatient and SNF services, which corresponded to an average annual Medicare savings of $190 million. There was greater functional improvement in HHVBP states than comparison states and no statistically significant change in emergency department use or most measures of patient experience.

**Conclusions and Relevance:**

In this cohort study, the HHVBP model was associated with lower Medicare payments that were associated with lower utilization of inpatient and SNF services, with better or similar quality of care.

## Key Points

**Question:**

How did quality, utilization, and Medicare payments differ after the 5 years of the Home Health Value-Based Purchasing (HHVBP) model?

**Findings:**

In this cohort study of US patients who received care at a home health agency between 2013 and 2020 in 9 original HHVBP states compared with those in comparison states, a difference-in-differences analysis found the HHVBP model was associated with lower Medicare payments that were associated with lower utilization of inpatient and skilled nursing facility services. Quality was better or similar.

**Meaning:**

The study results suggest that financial incentives for home health agency quality performance were associated with reduced Medicare payments and utilization while improving or maintaining quality.

## Teaser

This cohort study examines quality, utilization, and Medicare payments for home health patients in home health value-based purchasing model states compared with those in comparison states.

## Introduction

In January 2016, the Center for Medicare & Medicaid Innovation of the US Centers for Medicare & Medicaid Services (CMS) initiated the Home Health Value-Based Purchasing (HHVBP) model in 9 states: Arizona, Florida, Iowa, Massachusetts, Maryland, Nebraska, North Carolina, Tennessee, and Washington. These states were randomly selected by CMS from 9 regional groupings (eTable 1 in the Supplement), and all Medicare-certified home health agencies in these states were required to participate in the model.^1,2^

Medicare’s home health care benefit covers skilled nursing, physical, occupational, and speech therapies, aide services, and medical social work services to beneficiaries who need intermittent care and cannot leave their homes without considerable effort, with the goal of treating illness and injury so that patients can regain or maintain independence. A physician may initiate home health care as follow-up after a hospitalization or as a referral from the community (34% and 66% of initial home health episodes, respectively).^3^ Since 2001, home health services have been paid for under Medicare’s Home Health Prospective Payment System, which pays agencies a predetermined amount for each episode of care that is adjusted for case-mix, service use, geographic variation in wages, and other factors to account for episodes associated with especially low or high resource use. To support efforts to improve the delivery of health care and spend health care dollars more wisely, CMS designed the HHVBP model to improve the quality of home health care services by providing financial incentives to agencies for improvements in the quality of care they deliver.^1^ However, the HHVBP model does not include incentives to reduce overall use of home health care.

Within HHVBP, Medicare payments for fee-for-service (FFS) home health episodes are adjusted upward or downward based on an agency’s total performance score (TPS), a composite score of its quality achievement and improvement. To determine the payment adjustments for a given year, CMS calculates a TPS for each agency based on its scores for each of the performance measures achieved 2 years earlier. The HHVBP performance measures for 2020 are shown in Table 1. Agencies received points based either on their achievement level compared with baseline threshold values or improvement compared with their baseline performance; these points are calculated separately for each measure in each model state. The payment adjustments, which increased each year based on the original design of the model (Table 2), are intended to reward agencies with relatively higher achieved quality or improved quality compared with other agencies in their state and reduce payments to agencies with lower levels, such that they are budget neutral overall across agencies. The primary research objective of this study was to determine whether the HHVBP model was associated with improved quality and reduced health care utilization and Medicare payments for home health patients.

**Table 1.** HHVBP Performance Measures for 2020

Measure name	Measure type	Data source
ED use without hospitalization among first HH episodes	Utilization outcome	Medicare claims
Unplanned acute care hospitalization among first HH episodes	Utilization outcome	Medicare claims
Discharged to community	Outcome	OASIS
Improvement in dyspnea	Outcome	OASIS
Improvement in management of oral medications	Outcome	OASIS
Improvement in pain interfering with activity	Outcome	OASIS
TNC change in self-care^a^	Composite outcome	OASIS
TNC change in mobility^a^	Composite outcome	OASIS
How often the home health team gave care in a professional way (professional care)	Patient experience outcome	HHCAHPS
How well did the home health team communicate with patients (communication)?	Patient experience outcome	HHCAHPS
Did the home health team discuss medicines, pain, and home safety with patients (discussion of care)?	Patient experience outcome	HHCAHPS
How do patients rate the overall care from the home health agency (overall care)?	Patient experience outcome	HHCAHPS
Would patients recommend the home health agency to friends and family (likely to recommend)?	Patient experience outcome	HHCAHPS
Influenza vaccination coverage for home health care personnel^b^	Process	Agency self-report
Herpes Zoster (shingles) vaccination for patient^b^	Process	Agency self-report
Advance care plan^b^	Process	Agency self-report

Abbreviations: ED, emergency department; HH, home health; HHCAHPS, Home Health Consumer Assessment of Healthcare Providers and Systems; HHVBP, home health value-based purchasing; OASIS, Outcome and Assessment Information Set; TNC, total normalized composite.

^a^ These measures were added for 2019 and all subsequent years of the HHVBP model. They replaced 3 other OASIS-based measures: improvement in ambulation-locomotion, improvement in bathing, and improvement in bed transferring. Previous years of the HHVBP model also included 3 OASIS-based process measures: drug education on medications provided to patient/caregiver during episodes of care was dropped for performance year 2018 and all subsequent years, and the influenza immunization received for current flu season and pneumococcal polysaccharide vaccine ever received measures were dropped for performance year 2019 and all subsequent years.

^b^ Home health agencies in HHVBP states receive points for reporting these measures, but their performance on these measures does not affect their total performance score. These measures were only available for agencies in HHVBP states and not included in our analyses.

**Table 2.** Original HHVBP Model Maximum Payment Adjustment Amounts for Payment Years 1 Through 4 by Calendar Year

Calendar year	Payment adjustment?	Maximum payment adjustment
2016	No	NA
2017	No	NA
2018	Yes, based on 2016 TPS	±3%
2019	Yes, based on 2017 TPS	±5%
2020	Yes, based on 2018 TPS	±6%
2021	Yes, based on 2019 TPS	±7%

Abbreviations: HHVBP, home health value-based purchasing; NA, not applicable; TPS, total performance score.

## Methods

The original HHVBP Model design reflects (1) random selection by CMS of 9 states from 9 regional groupings (each containing 5 or 6 states) that were differentiated based on geographic location, utilization, demographic characteristics, and clinical characteristics^2^ and (2) mandatory participation of all Medicare-certified home health agencies in these selected states.^1^ By using observations of home health patients, our study examined how agencies in the 9 model states responded to the original HHVBP Model compared with agencies in nonmodel states. We examined all nonprocess measures used to derive agencies’ TPS (Table 1) as well as additional measures (eg, Medicare spending) to more fully evaluate the effects of the model. It was determined that this study was exempt from institutional review board approval and that patient consent was not needed owing to use of secondary data without identifiable information. This study included the required items from the Strengthening the Reporting of Observational Studies in Epidemiology (STROBE) reporting guideline.

### Data Sources

For this cohort study conducted in 2021, we used all nonmissing Medicare Part A and Part B enrollment and claims data from January 1, 2013, to March 31, 2021, that contained Medicare beneficiary demographic and enrollment characteristics, diagnoses, and service use. These data were linked with available Outcome and Assessment Information Set (OASIS) assessment data, which included demographic characteristics, levels of functioning, and other indicators of patient status for all Medicare and Medicaid home health patients. These were then linked to agency and county-level data to construct a home health episode–level analytic file. Home Health Consumer Assessment of Healthcare Providers and Systems (HHCAHPS) survey data were linked with Medicare data aggregated to the home health agency level to analyze patient experience.

### HHVBP Population

The HHVBP study population included all adult Medicare and Medicaid beneficiaries who had a home health episode that began between January 1, 2013, and December 31, 2020, in 1 of the 9 HHVBP states. Medicare FFS beneficiaries were included if they were continuously enrolled in Medicare Part A and met measure-specific requirements for coverage.^4^

### Comparison Group

The comparison group included all Medicare-certified agencies and adult Medicare and Medicaid beneficiaries with a home health episode that began during the same period in 1 of the 41 non-HHVBP states. The same comparison group approach was used for all analyses to facilitate interpretation of findings across the diverse outcomes that corresponded to different subpopulations of home health patients, involved different units of analysis, and were measured using different data sources. To avoid biased and imprecise effect estimates, a comparison population was defined that had agency and patient characteristics similar to the HHVBP population during the baseline period. The randomized selection of CMS of 9 HHVBP states and mandatory participation of all Medicare-certified agencies in these states helped guard against selection bias, and descriptive analyses suggested that the HHVBP states and non-HHVBP states had similar beneficiary, agency, and episode characteristics. As shown in eTable 2 in the Supplement, almost all of the covariates had an absolute value of the standardized difference in means before the intervention less than 0.25, which is a commonly used threshold for determining if differences in mean values between treatment groups are negligible.^5,6^ We used regression adjustment (discussed later) to control for differences between the 2 groups. A comparison of baseline mean values of outcomes revealed similar levels across HHVBP and non-HHVBP states, as absolute values of standardized differences in means for all such measures were substantially less than 0.25 (Table 3).

**Table 3.** Unadjusted Utilization, Medicare Payments, OASIS-Based Quality, and Patient Experience Measures for Home Health Patients

Measure (unit of analyses)	HHVBP states		Non-HHVBP states		Baseline standardized difference in means
	Baseline^a^	Intervention^a^	Baseline^a^	Intervention^a^	
HH agency-years, No.^b^	6906	9985	29 446	44 808	NA
HH FFS patients, No.^b^	2 525 841	4 014 649	7 898 642	12 570 221	NA
HH FFS episodes, No.^b^^,^^c^	4 422 930	7 704 805	15 417 548	26 353 991	NA
OASIS episodes, No.^b^	4 512 774	8 146 184	14 698 265	27 291 616	NA
HH utilization (county-year level)					
Percentage of FFS beneficiaries with at least 1 HH episode, county-year level, % mean (SD)	9.8 (416.1)	9.1 (332.1)	9.3 (262.2)	8.9 (242.4)	0.001
Health care utilization during HH episodes (FFS episode level)					
Unplanned acute care hospitalizations among first HH episodes, %	15.7	15.5	16.3	15.6	–0.017
Total ED use among first HH episodes, %	26.6	27.6	27.6	27.6	–0.022
Outpatient ED use among first HH episodes, %	11.7	12.5	12.3	12.6	–0.017
ED use followed by inpatient admission among first HH episodes, %	14.2	14.5	14.2	14.1	–0.002
SNF use among all HH episodes, %	4.9	4.4	4.0	3.7	0.040
Average Medicare Parts A and B payments per day (FFS episode level)					
During and following FFS HH episodes of care, mean (SD), $^d^	130.85 (1525.42)	152.80 (1502.31)	127.69 (1537.98)	153.84 (1518.61)	0.002
Components of average Medicare payments per day during and following FFS HH episodes of care^d^ (FFS episode level), mean (SD), $					
HH	38.44 (186.47)	44.08 (163.57)	36.38 (173.17)	45.90 (166.61)	0.011
Inpatient	43.93 (1189.95)	53.82 (1271.72)	45.97 (1239.92)	55.50 (1322.81)	–0.002
Outpatient institutional	10.81 (237.28)	14.24 (272.55)	11.56 (241.17)	15.35 (277.93)	–0.003
ED and observation stays	3.01 (88.48)	3.98 (110.26)	2.73 (79.94)	3.59 (99.65)	0.003
Other	7.71 (205.31)	10.17 (236.50)	8.75 (214.63)	11.67 (247.69)	–0.005
SNF	12.15 (432.37)	10.79 (365.33)	11.13 (421.55)	10.53 (384.83)	0.002
Hospice	3.20 (178.63)	4.32 (173.28)	2.60 (150.02)	3.76 (155.56)	0.003
Part B noninstitutional^e^	22.79 (286.90)	26.99 (292.76)	21.02 (276.72)	24.99 (282.07)	0.006
OASIS-based quality measures (OASIS episode level)					
Discharged to community, %	72.8	73.1	70.1	71.8	0.059
TNC change in self-care, mean (SD)	1.37 (1.12)	1.88 (1.19)	1.28 (1.13)	1.75 (1.20)	0.080
TNC change in mobility, mean (SD)	0.43 (0.41)	0.67 (0.46)	0.41 (0.42)	0.63 (0.47)	0.060
Improvement in dyspnea, %	66.7	81.5	66.1	78.9	0.011
Improvement in management of oral medications, %	51.5	71.5	53.9	69.9	–0.047
Improvement in pain interfering with activity, %	70.7	82.4	67.7	79.7	0.066
HHCAHPS-based patient experience measures (agency level), %^f^					
How often the home health team gave care in a professional way (professional care), agency % mean (SD)	88.8 (5.26)	88.4 (5.58)	88.2 (5.73)	88.0 (6.35)	0.111
How well did the home health team communicate with patients (communication), agency % mean (SD)	85.9 (6.09)	85.5 (6.42)	85.3 (6.32)	85.2 (7.07)	0.106
Did the home health team discuss medicines, pain, and home safety with patients (discussion of care), agency % mean (SD)	82.8 (7.10)	82.0 (7.90)	83.8 (7.08)	83.3 (7.87)	–0.130
How do patients rate the overall care from the home health agency (overall care), agency % mean (SD)	84.4 (8.30)	84.2 (8.37)	83.7 (9.12)	83.7 (10.10)	0.091
Would patients recommend the home health agency to friends and family (likely to recommend), agency % mean (SD)	79.6 (10.02)	78.7 (10.46)	78.4 (11.06)	77.7 (12.02)	0.119

Abbreviations: ED, emergency department; FFS, fee for service; HH, home health; HHCAHPS, Home Health Consumer Assessment of Healthcare Providers and Systems; HHVBP, home health value-based purchasing; OASIS, Outcome and Assessment Information Set; PDGM, Patient Driven Groupings Model; SNF, skilled nursing facility; TNC, total normalized composite.

^a^ Baseline period was from January 1, 2013, to December 31, 2015; the intervention period was from January 1, 2016, to December 31, 2020.

^b^ Reflects total number of observations across the baseline and intervention periods. The sample size for each measure may be lower than these totals because of missing data or measure-specific reporting requirements. See eTable 8 in the Supplement for analysis-specific sample sizes.

^c^ Home health episodes were defined using information reported on home health FFS claims, with the episode start date corresponding to the home health “claim from” date, and the episode end date corresponding to the home health “claim through” date.

^d^ Reflects Medicare payments during the home health episode through 37 days following the date of the last home health visit. This table reflects the Medicare spending post-PDGM values; pre-PDGM values showed a similar trend.^4^

^e^ Includes Part B carrier and durable medical equipment claims.

^f^ Mean percentages and standard deviations were calculated with agency-level data for the patient experience measures, reflecting the agency-level percentage of survey respondents reporting favorable experience in each domain. The overall care measure reflects the percentage of respondents who rated the agency favorably with a 9 or 10 on a 10-point scale.

### Claims-Based Outcomes

Claims-based measures correspond to Medicare FFS beneficiaries who received home health care. Unplanned acute care hospitalizations, emergency department (ED) visits (including outpatient only, inpatient, and total), and skilled nursing facility (SNF) use within 60 days of the start of the first home health episode were analyzed. Two of these outcomes (unplanned hospitalizations and outpatient ED use) were closely associated with the claims-based measures used in the HHVBP model^4^ and were part of the measure set used to determine each agency’s payment adjustment (Table 1). Average Medicare payments (which reflect the HHVBP payment adjustments) per day for FFS Medicare beneficiaries for all services covered under Medicare Parts A and B during the home health episode and up to 37 days after the last home health visit were also examined, as well as its components to understand of the factors associated with total payments for home health patients. To mitigate a potential source of bias because of differential change during the follow-up period between HHVBP and non-HHVBP states that was likely associated with the implementation by CMS of the Patient Driven Groupings Model, an alternative specification of spending measures was used in 2020 (eTable 3 in the Supplement).^7^

### OASIS-Based Outcomes

The OASIS-based measures that were part of the HHVBP measure set (Table 1) were also examined, including 2 total normalized composite (TNC) measures with a continuous range in normalized change values (–3 to 3 for mobility; –6 to 6 for self-care).^8,9^ Agencies are required to conduct a comprehensive assessment of all home health patients using the OASIS instrument.^10^

### Survey Outcomes

Finally, agency-level patient experience measures based on the HHCAHPS survey were also examined. All Medicare-certified agencies that have 60 or more HHCAHPS eligible patients a year must contract with an approved HHCAHPS survey vendor to administer the survey on their behalf and submit survey data to CMS.^11^

### Statistical Analysis

Multivariate linear regression within a difference-in-differences (D-in-D) framework was used to evaluate the effects of the model, comparing the changes observed in the 9 HHVBP states with those in the 41 comparison states based on data for the baseline period before HHVBP implementation (2013-2015) and cumulatively through the first 5 performance years of the model (2016-2020). We calculated the average annual estimate as a linear combination of the postimplementation year-specific effect estimates, weighted by the proportion of episodes or days or agency-year observations, as appropriate, in each year. The D-in-D design controlled for common changes to all home health patients over time, as well as for unmeasured differences between model and comparison states that did not change over time.^12,13^ The framework rests on the critical assumption that, in the absence of the HHVBP model, the outcomes in the 2 groups would have changed in a parallel manner over time. Positive (or negative) D-in-D estimates can be interpreted to mean that the HHVBP states had higher (or lower) measure values than estimated in the absence of the HHVBP model.

Several criteria were used to determine beneficiary, agency, and episode characteristic adjustments in the D-in-D models, including data availability across multiple populations of interest (eg, Medicare beneficiaries who receive home health care, all home health patients with Medicare or Medicaid coverage, and home health agencies); differential trends and degree of imbalance between HHVBP and non-HHVBP states during the baseline period; and the association with outcomes and correlation with other covariates. The assumption of parallel trends during the baseline period between the HHVBP and non-HHVBP states was tested with and without covariate adjustments using (1) placebo tests for effects in 2015 (expecting null effect) when the model was not yet implemented and (2) graphical plots of differences in baseline trends in unadjusted and adjusted outcomes, with slopes of plotted lines close to 0 indicative of measures moving in a parallel manner for the 2 groups. Beneficiary demographic characteristics, core clinical characteristics, and agency characteristics for episode-level measures were added to improve the D-in-D model’s ability to satisfy the parallel trends assumption for key measures of interest and establish valid inferences about the model’s effect. Given the onset of the COVID-19 public health emergency in 2020, analyses of 2020 data also included adjustments for current or recent beneficiary COVID-19 diagnoses reported in claims (for claims-based outcomes) and county-month–level rates of COVID-19 incidence and COVID-19–related hospitalizations among Medicare FFS beneficiaries (for all outcomes). A comparison of monthly reported COVID-19 diagnoses among FFS episodes during 2020 indicated similar trends in the HHVBP and comparison states (eFigure 1 in the Supplement).

For some outcomes (eg, FFS claims-based Medicare spending measures and OASIS-based outcomes) that showed evidence of nonparallel trends during the baseline period despite covariate adjustment, state-specific linear time trends were incorporated to control for remaining differences.^4^ In such cases, we assumed that outcomes in HHVBP and comparison states would continue along the same paths linearly absent the HHVBP model^14^; as such, the D-in-D reflected the deviation of the difference between the HHVBP and comparison group during the postperiod from the trend line. Analyses were performed using SAS, version 9.4 (SAS Institute), and statistical significance was set at *P *< .05.

## Results

Among the 34 058 796 home health episodes (16 584 870 beneficiaries) that began during the intervention period (2016-2020), 7 704 805 episodes (22.6%) were in HHVBP states and the remaining 26 353 991 (77.4%) were in non-HHVBP states (Table 3). The percentage of Medicare FFS beneficiaries with at least 1 home health episode declined slightly more in HHVBP states between the baseline period (2013-2015) and the intervention period (10.3% to 9.8%) compared with non-HHVBP states (9.8% to 9.6%). Unadjusted rates of unplanned hospitalizations among home health patients at baseline were lower in HHVBP states (15.7%) than in non-HHVBP states (16.3%), but converged to 15.5% and 15.6%, respectively, during the intervention. Unadjusted total ED use was also lower in HHVBP states at baseline (26.6% vs 27.6%), and converged toward levels in non-HHVBP states, increasing to 27.6% during the intervention while remaining unchanged in non-HHVBP states. Use of SNF at baseline was higher in HHVBP than non-HHVBP states (4.9% vs 4.0%), with similar declines in both groups during intervention (4.4% and 3.7%, respectively).

Average Medicare payments per day during and following home health episodes increased over time in HHVBP states ($130.85 to $152.80) and comparison states ($127.69 to $153.84). The components of total spending were similar in both groups, with inpatient services accounting for approximately one-third of Medicare payments, followed by home health services as the next largest component. Average dollar amounts increased in HHVBP and non-HHVBP states for all components except SNF (Table 3).

The unadjusted rate that home health patients were discharged to the community (vs an inpatient stay) increased between the baseline and intervention periods in HHVBP (72.8% to 73.1%) and non-HHVBP states (70.1% to 71.8%). For HHVBP and non-HHVBP states, the OASIS-based measures of functioning also increased, while agency performance on the 5 HHCAHPS-based measures remained high and relatively stable over time (Table 3).

The D-in-D analyses showed no differential change in the percentage of FFS beneficiaries with at least 1 home health episode between HHVBP states and the comparison group (Table 4). The HHVBP model was associated with a decline in unplanned hospitalizations of 0.15 percentage points (95% CI, –0.30 to –0.01; *P* = .04), a 1.0% decline compared with the baseline average of 15.7%. The other claims-based measure used in the HHVBP model (outpatient ED use) increased by 0.29 percentage points (95% CI, 0.16 to 0.42; *P* < .001) more in HHVBP states, a 2.5% increase compared with the baseline average of 11.7%. In contrast, HHVBP was associated with a decline in ED use, which was followed by an inpatient admission of 0.16 percentage points (95% CI, –0.30 to –0.02; *P* = .03), a 1.1% decrease compared with the baseline average of 14.2%. There was no differential association of the model with total ED use, which combines these 2 ED use measures. HHVBP was associated with a decline in SNF use of 0.34 percentage points (95% CI, –0.40 to –0.27; *P* = <.001), a 6.9% decrease compared with the baseline average of 4.9%.

**Table 4.** Cumulative Multivariate Results for Utilization, Medicare Payments, OASIS-Based Quality, and Patient Experience Measures for the HHVBP Model From 2016 to 2020

Measure	Difference-in-differences estimate, (95% CI)^a^	*P* value	Cumulative effect, %^b^
**HH utilization^c^**			
Percentage of FFS beneficiaries with at least 1 HH episode	0.17 (–0.16 to 0.50)	.31	1.7
**Health care utilization during HH episodes^c^**			
Unplanned hospitalizations among first HH episodes	–0.15 (–0.30 to –0.01)	.04	–1.0
Total ED use	0.13 (–0.06 to 0.31)	.18	0.5
Outpatient ED use	0.29 (0.16 to 0.41)	<.001	2.5
ED use followed by inpatient admission	–0.16 (–0.30 to –0.02)	.03	–1.1
SNF use	–0.34 (–0.40 to –0.27)	<.001	–6.9
**Average Medicare Parts A and B payments per day, $^c^**			
During and following FFS HH episodes of care^d^	–2.17 (–3.67 to –0.68)	.004	–1.6
**Components of average Medicare payments per day during and following FFS HH episodes of care, $** ^c^ ^,^ ^d^			
HH, $	–0.32 (–0.88 to 0.24)	.26	–0.7
Inpatient, $	–1.25 (–2.23 to –0.26)	.01	–2.8
Outpatient institutional, $	0.05 (–0.20 to 0.30)	.71	0.5
ED and observation stays	0.20 (0.10 to 0.29)	<.001	6.4
Other	–0.16 (–0.36 to 0.05)	.14	–2.1
SNF, $	–0.46 (–0.78 to –0.13)	.01	–4.0
Hospice, $	0.00 (–0.13 to 0.13)	.99	0.0
Part B noninstitutional^e^, $	–0.20 (–0.54 to 0.13)	.23	–0.9
**OASIS-based quality measures^c^**			
Discharged to community	0.91 (0.24 to 1.57)	.01	1.3
TNC change in self-care	0.04 (0.01 to 0.08)	.03	2.9
TNC change in mobility	0.01 (0.002 to 0.03)	.03	2.3
Improvement in dyspnea	–0.09 (–1.73 to 1.55)	.91	–0.1
Improvement in management of oral medications	2.49 (0.48 to 4.49)	.02	4.8
Improvement in pain interfering with activity	2.02 (0.70 to 3.34)	.003	2.9
**HHCAHPS-based patient experience measures**			
How often the home health team gave care in a professional way (professional care)	–0.21 (–0.43 to 0.01)	.06	–0.2
How well did the home health team communicate with patients (communication)?	–0.24 (–0.49 to 0.01)	.06	–0.3
Did the home health team discuss medicines, pain, and home safety with patients (discussion of care)?	–0.33 (–0.62 to –0.03)	.03	–0.4
How do patients rate the overall care from the home health agency (overall care)?	0.04 (–0.39 to 0.30)	.80	–0.05
Would patients recommend the home health agency to friends and family (likely to recommend)?	–0.01 (–0.42 to 0.40)	.95	–0.01

Abbreviations: D-in-D, difference-in-difference; ED, emergency department; FFS, fee-for-service; HH, home health; HHCAHPS, Home Health Consumer Assessment of Healthcare Providers and Systems; HHVBP, home health value-based purchasing; OASIS, Outcome and Assessment Information Set; PDGM, Patient Driven Groupings Model; SNF, skilled nursing facility; TNC, total normalized composite.

^a^ The D-in-D estimates are obtained from a regression model adjusted for beneficiary, agency, and episode-level characteristics along with state fixed effects (eTable 2 in the Supplement). Average FFS Medicare Parts A and B payments per day and their components and OASIS outcome measures were additionally adjusted for state linear trends. Underlying analytic sample sizes for these measures are included in eTable 7 in the Supplement. Detailed regression model results for claims-based measures, OASIS-based measures, and HHCAHPS-based measures are included in eTables 5, 6, and 7, respectively, in the Supplement.

^b^ The cumulative effect reflects the estimated change in HHVBP states compared with the comparison group during the first 5 years of the HHVBP model; negative values reflect decreases in utilization or Medicare savings.

^c^ Home health episodes were defined using information reported on home health FFS claims, with the episode start date corresponding to the home health “claim from” date, and the episode end date corresponding to the home health “claim through” date.

^d^ Reflects Medicare payments during the home health episode through 37 days following the date of the last home health visit. Estimates of the cumulative association with Medicare spending incorporate the pre-PDGM and post-PDGM approach.^4^

^e^ Includes Part B carrier and durable medical equipment claims.

The D-in-D analyses indicate a decline of $2.17 (95% CI, –$3.67 to –$0.68; *P* < .01) in average Medicare payments per day for FFS beneficiaries receiving home health services (Table 4), which corresponds to a 1.6% decline compared with average payments at baseline and reflects a reduction in Medicare payments during the home health episode rather than shortly after the episode ended (eTable 4 in the Supplement). Mirroring the changes observed in utilization, reductions in overall Medicare payments were primarily associated with reductions for inpatient services (–$1.25 per day; *P* = .01; 2.8% decline compared with average baseline spending) and SNF services (–$0.46 per day; *P* < .01; 4.0% decline compared with baseline). There was an estimated increase in average Medicare payments per day for outpatient ED visits and observation stays of $0.20 (95% CI, $0.10 to $0.29; *P* < .001), a 6.4% increase compared with baseline. A negative but nonsignificant change in Medicare payments for other outpatient institutional services offset higher Medicare payments for outpatient ED visits and observation stays such that there was no estimated association with payments for total outpatient institutional care (Table 4).

The D-in-D estimates indicated an association of HHVBP with modest improvements for most OASIS-based quality measures. There was an estimated increase in the percentage of home health patients discharged to the community of 0.90 percentage points (95% CI, 0.24 to 1.57; *P* < .01), a 1.3% increase compared with baseline. There were also larger improvements in the 2 composite measures of patient functional status in HHVBP states: 0.04 (95% CI, 0.01 to 0.08; *P* = .03) for the TNC change in self-care measure, reflecting a 2.9% increase compared with baseline, and 0.01 (95% CI, 0.002 to 0.03; *P* = .03) for the TNC change in mobility measure, reflecting a similar increase (2.3%) compared with baseline.

There were also larger estimated increases in HHVBP states for 2 of the other OASIS-based measures: a 4.8% increase in the percentage of patients showing improvement in management of oral medications compared with baseline, and a 2.9% increase in the percentage of patients showing improvement in pain compared with baseline (Table 4). In contrast to most other outcomes, there was a negative association of HHVBP with the discussion of care patient experience measure, with an estimated 0.33 percentage point decrease representing a 0.4% decrease from its baseline average of 82.8%. There was no statistically significant association of HHVBP with other patient experience measures (Table 4). Adjusted plots assessing parallel trends (critical for the robustness of D-in-D estimates) are included in eFigure 2 in the Supplement. Regression results for claims-based, OASIS-based, and HHCAHPS-based measures are included in eTables 5, 6, and 7 in the Supplement, respectively, and eTable 8 in the Supplement reports sample sizes.

## Discussion

This cohort study reports on the experience of home health patients and agencies through 2020, the fifth performance year of the HHVBP model and the third year that eligible agencies in HHVBP states received a payment adjustment. Findings across a range of measures of quality of care, health care utilization, and Medicare payments for home health patients suggest that HHVBP has been associated with changes that largely align with the goals of the model. Overall, results from D-in-D analyses indicate that the model is associated with a net decrease in utilization, slower growth in average Medicare spending, and improvements in certain aspects of quality among home health patients.

Regression results for average daily payments among FFS beneficiaries receiving home health services translated to an estimated average annual savings to Medicare of $190 million during 2016 to 2020 (range, $100.3 million to $262.9 million; Table 5^15^). During the first 5 years of the model, the largest contributor to overall savings for home health FFS patients was inpatient services; there was an estimated annual savings to Medicare of $109 million (range, $74.4 million to $159.4 million), which was followed by SNF services, with $40 million in estimated annual savings to Medicare. Savings to Medicare were partly offset by higher average annual Medicare payments for outpatient ED visits and observation stays in HHVBP states, with an annual average of $17 million (range, $11.6 million to $19.5 million). Agencies varied in their responses to HHVBP incentives, but overall, 4 of the 9 states showed evidence of savings.^4^

**Table 5.** Average Annual Medicare Payments Among FFS Home Health Beneficiaries From 2016-2020, Overall and Components^a^^,^^b^

Characteristic	Total Medicare Parts A and B payments during and following FFS home health episode of care, millions $				
	2016	2017	2018	2019	2020
**Overall**					
D-in-D estimate (95% CI), $^b^	–100.3 (–172.0 to –28.7)	–176.6 (–287.0 to –65.3)	–179.7 (–327.8 to –31.6)	–237.3 (–418.8 to –56.7)	–262.9 (–458.9 to –66.1)
* P* value	.01	.001	.02	.01	.01
Effect, %	–0.8	–1.4	–1.4	–1.9	–2.5
**Inpatient**					
D-in-D estimate (95% CI), $^b^	–74.4 (–125.4 to –22.4)	–96.2 (–170.4 to –23.0)	–102.1 (–198.7 to –5.4)	–159.4 (–278.9 to –39.8)	–114.5 (–242.7 to 12.9)
* P* value	.01	.01	.04	.01	.08
Effect, %	–1.8	–2.4	–2.5	–3.9	–3.2
**Outpatient ED and observation stays**					
D-in-D estimate (95% CI), $^b^	11.6 (6.3 to 16.1)	16.8 (9.7 to 23.0)	19.0 (9.0 to 28.9)	19.5 (8.0 to 31.0)	19.4 (7.3 to 32.3)
* P* value	<.001	<.001	<.001	.001	.002
Effect, %	4.1	6.1	6.7	7.0	8.0
**Skilled nursing facility**					
D-in-D estimate (95% CI), $^b^	–27.8 (–43.9 to –10.8)	–41.5 (–65.3 to –17.7)	–47.9 (–79.5 to –15.4)	–56.7 (–93.9 to –18.6)	–28.2 (–72.6 to 16.1)
* P* value	.001	<.001	.003	.003	.21
Effect, %	–2.7	–4.1	–4.7	–5.6	–2.9
**Home health**					
D-in-D estimate (95% CI), $^b^	12.5 (–10.8 to 34.9)	–19.4 (–59.2 to 20.3)	6.3 (–48.8 to 61.4)	5.3 (–63.8 to 75.3)	–150.0 (–226.6 to –73.4)
* P* value	.29	.34	.82	.87	<.001
Effect, %	0.3	–0.5	0.2	0.1	–4.8

Abbreviations: D-in-D, difference-in-difference; ED, emergency department; FFS, fee-for-service; OASIS, Outcome and Assessment Information Set.

^a^ Reflects Medicare payments during the home health episode through 37 days following the date of the last home health visit. Home health episodes were defined using information reported on home health FFS claims, with the episode start date corresponding to the home health “claim from” date, and the episode end date corresponding to the home health “claim through” date.

^b^ The D-in-D estimates were obtained from a regression model adjusted for beneficiary, agency, and episode-level characteristics along with state fixed effects (eTable 2 in the Supplement). Average FFS Medicare Parts A and B payments per day and their components and OASIS outcome measures were additionally adjusted for state linear trends.

For all but 2 of the HHVBP measures (outpatient ED use and discussion of care) that comprised the TPS (Table 1), HHVBP was associated either with changes in the intended direction or with no change. Results suggest that there were fewer unplanned hospitalizations and SNF admissions in HHVBP states and no statistically significant association with overall ED use. Because home health entails monitoring patient status, facilitating early interventions, and promoting rapid recovery of health and functional status, these adjusted claims-based measures of utilization may be interpreted as indicators of the quality of home health care in that higher quality care may result in fewer hospitalizations or admissions to SNFs. Agencies providing early and intensive home health services and improving medication and pain management are mechanisms by which HHVBP may contribute to reduced unplanned health care utilization and Medicare payments.^7^ Although statistically significant, the HHVBP association with discussion of care corresponds to only a 0.4 percentage relative decrease in the baseline value of 82.8% (Table 3), which does not suggest a meaningful association of HHVBP with patient experience with care. There was a positive association between HHVBP and most of the OASIS-based outcomes, including a relative increase in discharge to the community and functional status, and their effects are similar to other measures examined (Table 4). These findings also align with what agencies expressed in interviews from prior years that quality improvement efforts for OASIS assessments were a central focus.^16,17,18^ These findings also align with evidence reported by other researchers of increases in OASIS-based measures and agency star ratings associated with HHVBP model implementation.^19,20^ Because OASIS-based measures are not limited to Medicare FFS beneficiaries, performance changes may diffuse across the entire home health population rather than occurring only for Medicare FFS beneficiaries for whom agencies receive the performance-based HHVBP payment adjustments. However, socioeconomic vulnerabilities of some populations, such as those covered by Medicaid, may still present challenges to quality improvement.^7^

Changes in the HHVBP model design, such as the increasing magnitude of potential future payment adjustments, did not produce substantially different effects in later years (ie, 2018-2020) compared with its early years (ie, 2016-2017) across most measures.^4^ The limited exceptions include improvement in dyspnea and the percentage of patients discharged to community (which HHVBP has been associated with larger increases in later years), and SNF use and 2 HHCAHPS measures (discussion of care and professional care), which have larger declines in HHVBP during the later years.^4^

This study’s findings for HHVBP are distinct from studies of other value-based purchasing initiatives that have not demonstrated a strong association with improvements in quality of care or patient outcomes for hospitals^21,22,23,24^ or nursing homes.^25^ The wider range for potential payment adjustments (ie, up to 6% in 2020; Table 2) for HHVBP compared with 2% in the CMS hospital VBP program may be one reason for differences in findings. Although the average payment adjustment across all agencies in HHVBP states in 2020 was near 0 (−0.07%), with nearly half (48.5%) receiving a positive payment adjustment, almost 1 in 8 agencies (13%) received a payment adjustment greater than 2% (ie, 2% to 6%), and 1 in 6 (16%) received a payment adjustment less than –2% (ie, –6% to –2%).^26^ Other factors contributing to differences across VBP programs may stem from the complexity of services provided in hospitals compared with home health, which are associated with greater challenges for implementing mechanisms for quality improvement in hospitals compared with home health agencies. The lack of downside risk faced by clinicians in the nursing home VBP program may also account for the differences in findings.

Determining whether agencies with specific characteristics were primarily responsible for the savings and quality improvement attributed to the HHVBP model remains a topic for future investigation. Research by Mukamel and colleagues^27^ suggests that agencies with lower quality at baseline face net savings and costs that provide stronger incentives to improve quality compared with agencies with higher quality at baseline. Investigating whether agencies with lower baseline quality realized greater improvement in response to the model may provide further insight into mechanisms by which HHVBP is associated with outcomes. The study conclusions are strengthened by the HHVBP model design (randomized selection of states and mandatory participation of agencies in the selected states) and a D-in-D approach, with covariate adjustments to account for any observed imbalances or lack of parallel trends.

### Limitations

A potential study limitation is that the beneficiary and agency characteristics used in the multivariate regressions may not account for all relevant differences between HHVBP and non-HHVBP states that may have emerged postintervention. However, trends in beneficiary and agency characteristics do not suggest systematic changes over time between the 2 groups that differ substantially and were not already under way before HHVBP. Additionally, conducting statistical testing on multiple outcomes increases the likelihood of falsely rejecting the null hypothesis across outcomes, and standard errors were not adjusted to account for multiple comparisons. However, the strong pattern of observed associations for HHVBP that are consistent among associated outcomes and have been relatively persistent over time^16,17,18,26^ do not suggest that this is a risk in forming conclusions about the effect of HHVBP.

## Conclusions

In this cohort study that examined the first 5 years of the original HHVBP model, we found evidence of a decline in Medicare spending, unplanned hospitalizations, and use of SNFs among FFS beneficiaries receiving home health services in HHVBP states, with no association with overall ED use. Improvements in quality measures were either greater or similar among home health patients in HHVBP states than those in comparison states. In November 2021, CMS finalized plans to expand the original HHVBP model nationally in January 2023, with 2022 serving as a preimplementation year, and ended the original HHVBP model 1 year early in December 2021.^15^ Additional analyses for the final year of the original HHVBP model in which the largest potential Medicare payment adjustments are applied (2021) are needed to assess longer-term effects of the model and further inform CMS as the model is expanded nationally.

## Notes

Supplement. **eTable 1.** HHVBP States and Non-HHVBP States in their Corresponding Regional Group **eTable 2.** Core Set of Covariates Used in Episode-Level Analyses for FFS Claims and OASIS Measures During Baseline and Intervention Periods, by HHVBP and non-HHVBP **eTable 3.** Measure specifications for Average Medicare Spending per Day during and following FFS Home Health Episodes of Care **eTable 4. **Average Medicare Parts A and B Payments Per Day, During and Following Home Health **eTable 5. **Multivariate D-in-D Regression Results for the Claims-based Measures **eTable 6.** Multivariate D-in-D Regression Results for the OASIS-based Measures **eTable 7.** Multivariate D-in-D Regression Results for the HHCAHPS-based Measures **eTable 8.** Sample Sizes for Analyses of Utilization, Medicare Payments, OASIS-based Quality, and HHCAHPS-based Measures **eFigure 1.** Percentage of Home Health Episodes with an Initial COVID-19 Diagnosis, January to December 2020 **eFigure 2.** Assessing Parallel Trends for Outcomes based on Adjusted Models
