# Supplementary material for: Association of the Home Health Value-Based Purchasing Model With Quality, Utilization, and Medicare Payments After the First 5 Years
Source: JAMA Health Forum. 2022 Sep 23;3(9):e222723. doi: 10.1001/jamahealthforum.2022.2723 (PMC9508657; doi:10.1001/jamahealthforum.2022.2723)
Supplement: Supplement. — eTable 1. HHVBP States and Non-HHVBP States in their Corresponding Regional Group eTable 2. Core Set of Covariates Used in Episode-Level Analyses for FFS Claims and OASIS Measures During Baseline and Intervention Periods, by HHVBP and non-HHVBP eTable 3. Measure specifications for Average Medicare Spending per Day during and following FFS Home Health Episodes of Care eTable 4. Average Medicare Parts A and B Payments Per Day, During and Following Home Health eTable 5. Multivariate D-in-D Regression Results for the Claims-based Measures eTable 6. Multivariate D-in-D Regression Results for the OASIS-based Measures eTable 7. Multivariate D-in-D Regression Results for the HHCAHPS-based Measures eTable 8. Sample Sizes for Analyses of Utilization, Medicare Payments, OASIS-based Quality, and HHCAHPS-based Measures eFigure 1. Percentage of Home Health Episodes with an Initial COVID-19 Diagnosis, January to December 2020 eFigure 2. Assessing Parallel Trends for Outcomes based on Adjusted Models [file jamahealthforum-e222723-s001.pdf]

Supplemental Online Content

Pozniak A, Lammers E, Mukhopadhyay P, et al. Association of the home health value-based purchasing model with quality, utilization, and Medicare payments after the first 5 years. *JAMA Health Forum*. 2022;3(9):e222723. doi:10.1001/jamahealthforum.2022.2723

eTable 1. HHVBP States and Non-HHVBP States in their Corresponding Regional Group

eTable 2. Core Set of Covariates Used in Episode-Level Analyses for FFS Claims and OASIS Measures During Baseline and Intervention Periods, by HHVBP and non-HHVBP

eTable 3. Measure specifications for Average Medicare Spending per Day during and following FFS Home Health Episodes of Care

eTable 4. Average Medicare Parts A and B Payments Per Day, During and Following Home Health

eTable 5. Multivariate D-in-D Regression Results for the Claims-based Measures

eTable 6. Multivariate D-in-D Regression Results for the OASIS-based Measures

eTable 7. Multivariate D-in-D Regression Results for the HHCAHPS-based Measures

eTable 8. Sample Sizes for Analyses of Utilization, Medicare Payments, OASIS-based Quality, and HHCAHPS-based Measures

eFigure 1. Percentage of Home Health Episodes with an Initial COVID-19 Diagnosis, January to December 2020

eFigure 2. Assessing Parallel Trends for Outcomes based on Adjusted Models

This supplemental material has been provided by the authors to give readers additional information about their work.

© 2022 Pozniak A et al. *JAMA Health Forum.*

eTable 1. HHVBP States and Non-HHVBP States in their Corresponding Regional Group

HHVBP State	Non-HHVBP States in Regional Group
Arizona	New Mexico, California, Nevada, Utah, Colorado
Florida	Texas, Oklahoma, Louisiana, Mississippi
Iowa	North Dakota, South Dakota, Montana, Wisconsin, Minnesota
Massachusetts	Vermont, Maine, Connecticut, Rhode Island, New Hampshire
Maryland	Delaware, New Jersey, Pennsylvania, New York
North Carolina	Alabama, Georgia, South Carolina, Virginia
Nebraska	Ohio, West Virginia, Indiana, Missouri, Kansas
Tennessee	Illinois, Kentucky, Arkansas, Michigan
Washington	Oregon, Alaska, Hawaii, Wyoming, Idaho

Abbreviations: HHVBP, home health value-based purchasing.

© 2022 Pozniak A et al. *JAMA Health Forum.*

eTable 2. Core Set of Covariates Used in Episode-Level Analyses for FFS Claims and OASIS Measures During Baseline and Intervention Periods, by HHVBP and non-HHVBP

**Covariate [a]**	**HHVBP States**		**Non-HHVBP States**		**Standardized Difference in Means [b]**	
	**Baseline [c]**	**Intervention [c]**	**Baseline [c]**	**Intervention [c]**	**Baseline [c]**	**Intervention [c]**
HH FFS episodes, no. [d]	4,442,930	7,704,805	15,417,548	26,353,991	N/A	N/A
**Beneficiary Characteristics**						
Age						
<65 years	13.0%	11.2%	15.6%	13.7%	-0.08	-0.08
65-84 years	56.3%	57.4%	56.8%	58.4%	-0.01	-0.02
85 years and older	30.8%	31.4%	27.6%	27.8%	0.07	0.08
Sex						
% Male	37.9%	39.6%	37.2%	39.0%	0.01	0.01
% Female	62.1%	60.4%	62.8%	61.0%	-0.01	-0.01
Race/Ethnicity (Mutually Exclusive) [e]						
Hispanic (regardless of Black/White/Other race)	9.5%	6.1%	8.5%	7.5%	0.03	-0.06
Non-Hispanic Black	10.1%	10.0%	16.5%	13.6%	-0.21	-0.12
Non-Hispanic White	79.1%	82.4%	71.8%	75.2%	0.18	0.19
Non-Hispanic other	1.2%	1.4%	3.0%	3.5%	-0.17	-0.17
Non-Hispanic multiracial	0.1%	0.1%	0.2%	0.2%	-0.02	-0.02
% Dual eligible	30.3%	25.0%	34.5%	32.3%	-0.09	-0.17
% Rural	4.9%	5.0%	9.4%	9.2%	-0.21	-0.20
% of persons in the patient’s county of residence who are ages 25 years and older with less than a high school diploma	12.6%	12.2%	14.9%	14.6%	-0.48	-0.52
Alignment with selected APMs						
BPCI Model 2	0.9%	1.6%	0.9%	1.4%	0.01	0.02
BPCI Model 3	0.1%	0.3%	0.2%	0.2%	-0.02	0.01
BPCI Advanced	0.0%	0.7%	0.0%	0.8%	*	-0.01

© 2022 Pozniak A et al. *JAMA Health Forum.*

Covariate [a]	HHVBP States		Non-HHVBP States		Standardized Difference in Means [b]	
	**Baseline [c]**	**Intervention [c]**	**Baseline [c]**	**Intervention [c]**	**Baseline [c]**	**Intervention [c]**
CJR	0.0%	0.6%	0.0%	0.6%	*	-0.01
MSSP	18.2%	21.0%	14.7%	20.2%	0.09	0.02
Next Generation ACO	0.0%	4.8%	0.0%	2.3%	*	0.11
OCM	0.0%	1.6%	0.0%	1.6%	*	0.001
Pioneer ACO	4.5%	0.7%	2.0%	0.1%	0.12	0.07
**Agency Characteristics**						
Ownership						
For-profit	71.0%	72.3%	69.5%	72.2%	0.03	0.002
Non-profit	25.9%	25.3%	28.0%	26.1%	-0.05	-0.02
Government-owned	3.1%	2.4%	2.5%	1.7%	0.04	0.05
Setting						
Hospital-based	8.4%	7.5%	11.1%	9.2%	-0.10	-0.06
Freestanding	91.6%	92.5%	88.9%	90.8%	0.10	0.06
Chain Affiliation						
Affiliated	48.7%	53.6%	34.4%	39.2%	0.29	0.29
Not Affiliated	42.3%	37.8%	57.8%	54.0%	-0.31	-0.33
Missing	3.1%	0.9%	2.1%	0.8%	0.06	0.01
Undetermined	5.9%	7.8%	5.7%	6.0%	0.01	0.07
HHA Age						
<4 years	7.3%	2.2%	6.8%	3.8%	0.02	-0.11
4-10 years	25.4%	18.2%	24.1%	17.8%	0.03	0.01
>10 years	67.3%	79.6%	69.1%	78.4%	-0.04	0.03
HHA Size						
1-59 OASIS episodes	1.3%	0.8%	3.4%	2.4%	-0.19	-0.19
60-249 OASIS episodes	7.5%	4.9%	15.6%	12.4%	-0.31	-0.35
250-499 OASIS episodes	11.1%	8.0%	14.7%	12.4%	-0.11	-0.16
500-999 OASIS episodes	17.1%	14.7%	16.8%	16.4%	0.01	-0.05

© 2022 Pozniak A et al. *JAMA Health Forum.*

Covariate [a]	HHVBP States		Non-HHVBP States		Standardized Difference in Means [b]	
	Baseline [c]	Intervention [c]	Baseline [c]	Intervention [c]	Baseline [c]	Intervention [c]
1,000 or more OASIS episodes	63.0%	71.5%	49.5%	56.4%	0.28	0.34
Core Clinical Indicators Used for Episode-Level Impact Measures [f]						
Ambulation and Locomotion						
Able to independently walk with the use of a one-handed device	13.9%	6.2%	15.1%	7.1%	-0.04	-0.03
Requires two-handed device for level ground or human assistance for stairs and uneven ground	32.3%	15.6%	32.8%	19.4%	-0.01	-0.10
Walks only with supervision or assistance from another at all times	42.6%	64.0%	40.2%	59.7%	0.05	0.09
Chairfast to bedfast	11.2%	14.1%	11.9%	13.8%	-0.02	0.01
Risk for Hospitalization						
Multiple hospitalizations in past 6 months	36.4%	33.2%	37.1%	33.2%	-0.01	-0.001
History of falls	32.8%	36.0%	31.4%	33.5%	0.03	0.05
Currently taking 5 or more medications	88.3%	92.5%	87.0%	92.1%	0.04	0.02
Surgical wound	24.4%	25.4%	25.6%	26.7%	-0.03	-0.03
Requires urinary catheter	4.4%	4.7%	4.6%	4.7%	-0.01	-0.002
Discharged from inpatient facility in last 14 days	67.8%	68.7%	71.4%	70.8%	-0.08	-0.05
Pressure ulcer						
Pressure ulcer stage 2	3.2%	3.0%	3.4%	3.2%	-0.01	-0.01
Pressure ulcer stage 3	0.9%	0.9%	1.0%	1.0%	-0.01	-0.01
Pressure ulcer stage 4	0.5%	0.5%	0.6%	0.6%	-0.01	-0.01
Pressure ulcer not stageable	0.9%	1.0%	1.1%	1.1%	-0.01	-0.01
Neoplasm diagnosis	8.1%	8.8%	8.7%	9.1%	-0.02	-0.01

Abbreviations: ACO, accountable care organization; APM, alternative payment model; BPCI, bundled payment for care improvement; CJR, comprehensive care for joint replacement; FFS, fee-for-service; HHA, home health agency; HH, home health; HHCAHPS, Home Health Care

© 2022 Pozniak A et al. *JAMA Health Forum.*

Consumer Assessment of Healthcare Providers and Systems; HHVBP, home health value-based purchasing; MSSP, Medicare shared savings program; OASIS, Outcome and Assessment Information Set; OCM, oncology care model.

[a] For the HHA-level HHCAHPS measures, we excluded patient age and area education variables since comparable factors were already accounted for in the risk adjusted HHCAHPS measure values. Additionally, we did not specify OASIS episode characteristics (aggregated to the agency level) as covariates for analyses of the HHA-level impact measures, given that each of these measure values already reflected risk adjustment based on any clinical factors that were deemed relevant to measuring agency performance under HHVBP.

[b] Standardized differences in means compare the means of HHVBP and non-HHVBP states during each time period—baseline (2013 – 2015) and intervention (2016 – 2020). In this case, the standardized difference in means is the difference in means between the HHVBP and non-HHVBP groups divided by the standard deviation of the HHVBP group.

[c] Baseline period was from January 1, 2013, to December 31, 2015; the intervention period was from January 1, 2016, to December 31, 2020.

[d] Reflects total number of observations across the baseline and intervention periods. The sample size for each measure may be lower than these totals due to missing data or measure-specific reporting requirements.

[e] Race indicators (white, Black, Hispanic, other race) came from OASIS assessment item M0140 and was recoded to mutually exclusive groups; American Indian or Alaska Native, Asian and Native Hawaiian or Pacific Islander are grouped into the “other” race category.

[f] Derived from OASIS assessment at start of home health care.

© 2022 Pozniak A et al. *JAMA Health Forum.*

eTable 3. Measure specifications for Average Medicare Spending per Day during and following FFS Home Health Episodes of Care

**Measure Description**	Pre-PDGM<br>Average Medicare Part A and Part B payments (or "Expenditure Components" listed below) per day during and up to 37 days following HH episodes of care. This measure includes payments that occur between the start of the HH episode (start of care [SOC]) and a 37-day look-out period following the last HH visit (end of care) or until the start of the next HH episode that begins on or before the 37th day or until death, or loss of FFS Part A eligibility; whichever comes earlier. The length of the look-out period (37-day) is composed of 7 days post last HH visit and additional 30 days thereafter or until the start of the next HH episode that begins on or before the 37th day or until death or loss of FFS Part A eligibility; whichever comes earlier.<br>Post-PDGM:<br>Average Medicare Part A and Part B payments (or "Expenditure Components" listed below) per day during and up to 30 days following HH episodes of care. This measure includes payments that occur between the start of the HH episode (start of care [SOC]) and the 59th day after the SOC, the start of the next HH episode, death, or loss of FFS Part A eligibility; whichever comes earlier.
**Measure Numerator**	Pre-PDGM:<br>Total Medicare Part A and Part B payments (or "Expenditure Components" listed below) between the SOC and a 37-day look-out period following the last HH visit (end of care) or until the start of the next HH episode that begins on or before the 37th day or until death or loss of FFS Part A eligibility; whichever comes earlier.<br>Post-PDGM:<br>Total Medicare Part A and Part B payments (or "Expenditure Components" listed below) between the SOC and the 59th day after the SOC, the start of the next HH episode, death, or loss of FFS Part A eligibility; whichever comes earlier.
**Numerator Details**	Pre-PDGM:<br>Sum of Medicare payments on all Part A and Part B claims (or "Expenditure Components" listed below) with a claim start date (i.e., based on "CLM_FROM_DT") occurring between the SOC and a 37-day look-out period following the last HH visit (end of care) or until the start of the next HH episode that begins on or before the 37th day or until death or loss of FFS Part A eligibility; whichever comes earlier. Where applicable, the 37-day look-out period following the end of care is truncated to prevent possible double counting of payments for claims that occur during a subsequent HH episode beginning during this 37-day period.<br>Post-PDGM:<br>Sum of Medicare payments on all Part A and Part B claims (or "Expenditure Components" listed below) with a claim start date (i.e., based on "CLM_FROM_DT") occurring between the SOC and

© 2022 Pozniak A et al. *JAMA Health Forum.*

	the 59th day after the SOC, the start of the next HH episode, death, or loss of FFS Part A eligibility; whichever comes earlier. Where applicable, the look-out period is truncated to prevent possible double counting of payments for claims that occur during a subsequent HH episode beginning during this period.
**Measure Denominator**	Pre-PDGM:<br>Total number of eligible days accrued during and in periods of up to 37 days following the last HH visit date of all HH FFS episodes starting in a given calendar quarter.<br>Post-PDGM:<br>Total number of eligible days accrued during and in periods of up to 30 days following all HH FFS episodes starting in a given calendar quarter.
**Denominator Details**	Pre-PDGM:<br>Denominator includes all days occurring between the SOC and a 37-day look-out period following the last HH visit (end of care) or until the start of the next HH episode that begins on or before the 37th day or until death, or loss of FFS Part A eligibility, for HH episodes of all types (LUPAs, outliers, PEPs, etc.) starting in a given calendar quarter, irrespective of whether they are first, second, or higher episodes in a sequence. HH episodes are excluded in the absence of a HH visit date. The maximum number of days that can be included in the denominator is 97 days for a 60-day episode of care (60 + 37 days), unless the last HH visit date occurs before the HH episode claim end date and/or a subsequent HH episode, death, or loss of FFS Part A eligibility occurs prior to the end of the 37-day look–out period.<br>Post-PDGM:<br>Denominator includes all days occurring between the SOC and the 59th day after the SOC, the start of the next HH episode, death, or loss of FFS Part A eligibility, for HH episodes of all types (LUPAs, outliers, PEPs, etc.) starting in a given calendar quarter, irrespective of whether they are first, second, or higher episodes in a sequence. HH episodes are excluded in the absence of a HH visit date. The maximum number of days that can be included in the denominator is 60 days for a 30-day episode of care (30 + 30 days), unless a subsequent HH episode, death, or loss of FFS Part A eligibility occurs prior to the end of the 30-day episode or 30-day look-out period.
**Expenditure Components**	Besides the total Medicare Part A and Part B spending measure, we repeated the same calculation for each individual expenditure component, including: Medicare Part B carrier and DME combined, HH, Hospice, Inpatient, Outpatient Emergency Department and Observation Stays, other Outpatient, Outpatient types combined, and SNF and obtained average Medicare spending per day during and following FFS HH episodes of care for each of the expenditure components.
**Notes:**	This measure along with each of its components were individually capped at both the lower and upper ends to reduce the influence of extreme expenditure outliers. For each year, the measure was capped at the 99th percentile of the unweighted spending per-day measure (i.e. any values

© 2022 Pozniak A et al. *JAMA Health Forum.*

	greater than 99th percentile were set to the 99th percentile value), and any negative payment values were set to zero dollars. The post-PDGM spending measure definition was applied retrospectively to 60-day HH episodes from 2013-19 (all Medicare payments between the SOC and the 89th day after the SOC, the start of the next HH episode, death, or loss of FFS Part A eligibility; whichever comes earlier) in order to draw accurate inferences about the impacts in 2020 following the implementation of the PDGM.

Abbreviations: DME, durable medical equipment; FFS, fee-for-service; HH, home health; LUPA, low utilization payment adjustment; PDGM, Patient Driven Groupings Model; PEP, partial episode payment; SNF, skilled nursing facility

© 2022 Pozniak A et al. *JAMA Health Forum.*

eTable 4. Average Medicare Parts A and B Payments Per Day, During and Following Home Health

**Unadjusted Measure Values**	**HHVBP States**		**Non-HHVBP States**	
	**Baseline [a]**	**Intervention [a]**	**Baseline [a]**	**Intervention [a]**
Average Medicare parts A and B payments per day during FFS HH episodes of care (SD)	$150.60 ($1302.27)	2016-2019: $161.70 ($1416.21)	$135.34 ($1326.86)	2016-2019: $150.38 ($1443.50)
	$144.25 ($1412.40)	2020: $165.09 ($1367.15)	$135.79 ($1429.97)	2020: $162.43 ($1377.60)
Average Medicare parts A and B payments per day following FFS HH episodes of care [b] (SD)	$105.97 ($1303.35)	2016-2019: $114.93 ($1382.44)	$116.54 ($1411.44)	2016-2019: $123.51 ($1481.86)
	$82.25 ($1086.45)	2020: $113.31 ($1472.70)	$89.38 ($1167.71)	2020: $120.26 ($1574.35)
**Cumulative (2016-2020) Multivariate Results**	**D-in-D Estimate**	**(95% CI)**	**P-value**	**Cumulative Impact**
Average Medicare parts A and B payments per day during FFS HH episodes of care	-$2.07	(-$3.80, $0.34)	0.02	-1.4%
Average Medicare parts A and B payments per day following FFS HH episodes of care [b]	$0.08	(-$1.97, $2.12)	0.94	0.1%

Abbreviations: CI, confidence interval; D-in-D, difference-in-differences; FFS, fee-for-service; HH, home health; HHVBP, home health value-based purchasing; SD, Standard Deviation.

[a] Baseline period was from January 1, 2013, to December 31, 2015; the intervention period was from January 1, 2016, to December 31, 2020.

[b] Reflects Medicare payments during the home health episode through 37 days following the date of the last home health visit.

© 2022 Pozniak A et al. *JAMA Health Forum.*

eTable 5. Multivariate D-in-D Regression Results for the Claims-based Measures

	Health Care Utilization During HH Episodes			Combined Spending Model [a]	
	Unplanned acute care hospitalizations among 1st HH episodes	Outpatient ED use among 1st HH episodes	Skilled Nursing Facility Use	Pre-PDGM	Post-PDGM
N	21,003,491 FFS Episodes	21,003,491 FFS Episodes	43,759,916 FFS Episodes	93,641,792 FFS Episodes	
Intercept	3.52**	11.29**	2.44**	77.75**	-11.5**
State					
Alaska	0.47	2.01**	-2.73**	14.25**	-3.69
Alabama	1.6**	-3.4**	-0.97**	-27.09**	3.93**
Arkansas	1.16**	-1.86**	-0.84**	-21.88**	4.11**
Arizona	0.73*	-0.91*	-0.72**	11.85**	-3.27**
California	0.52	-2.69**	-0.34*	25.69**	-0.44
Colorado	-1.4**	-0.3	0.03	-7.73*	-2.28**
Connecticut	2.47**	-1.58**	2.25**	21.22**	-0.04
Delaware	1.48**	-2.4**	0.06	12.36**	-3.4**
Florida	1.76**	-4.23**	-0.45**	6.58*	0.11
Georgia	1.69**	-1.58**	-1.26**	-10.65**	2.35**
Hawaii	1.03**	2.03**	-0.84**	18.68**	-3.13**
Iowa	1.87**	-1.82**	0.78**	-6.86	-0.37
Idaho	-1.46**	-0.62	-1.02**	-18.33**	1.95*
Illinois	1.95**	-3.44**	-0.05	-2.54	3.32**
Indiana	2.25**	-1.59**	0.47**	0.39	3.07**
Kansas	2.05**	-2.28**	0.77**	-8.57**	1.8*
Kentucky	2.31**	-1.96**	-0.02	-14.22**	3.1**
Louisiana	0.92**	-1.5**	-2.16**	-23.35**	4.96**
Massachusetts	2.19**	-2.9**	1.05**	17.12**	0.45
Maryland	1.57**	-3.12**	0.09	23.21**	-1.66
Maine	0.78*	0.06	0.58**	-9.84**	0.85
Michigan	2.18**	-2.71**	-0.002	-1.22	0.16
Minnesota	1.83**	-2.34**	0.92**	0.64	-1.87*
Missouri	2.08**	-2.47**	0.15	-8.67**	-0.44

© 2022 Pozniak A et al. *JAMA Health Forum.*

	Health Care Utilization During HH Episodes			Combined Spending Model [a]	
	Unplanned acute care hospitalizations among 1st HH episodes	Outpatient ED use among 1st HH episodes	Skilled Nursing Facility Use	Pre-PDGM	Post-PDGM
Mississippi	0.52	-2.53**	-1.43**	-29.49**	5.28**
Montana	-0.39	-2.09**	-0.01	-19.05**	-0.5
North Carolina	1.1**	-1.43**	-0.66**	-12.65**	0.32
North Dakota	2.12**	-2.88**	0.94**	-8.24	-0.19
Nebraska	1.33**	-3.76**	0.77**	-1.91	-0.6
New Hampshire	1.66**	-0.9	0.9**	-2.3	0.57
New Jersey	2.61**	-4.5**	1.6**	26.47**	-1.65
New Mexico	-0.99**	0.11	-1.25**	-22.98**	3.8**
Nevada	0.77*	-2.32**	-1.04**	14.14**	1.81*
New York	2.87**	-4.5**	0.03	26.67**	-1.44
Ohio	1.95**	-1.47**	0.38*	-7.38*	2.57**
Oklahoma	0.61*	-0.77	-1.67**	-27.39**	5.36**
Oregon	-0.76*	0.61	-1.41**	-14.62**	2.06**
Pennsylvania	2.6**	-2.96**	0.51**	1.51	0.41
Rhode Island	2.01**	-2.03**	1.6**	6.81	0.64
South Carolina	0.84*	-1.76**	-1.17**	-12.67**	0.94
South Dakota	1.55**	-3.26**	1.4**	-6.88	-1.95
Tennessee	1.41**	-2.91**	-0.63**	-18.5**	3.46**
Texas	0.54	-1.96**	-1.5**	-13.88**	4.61**
Utah	-2.43**	-2.25**	-0.51**	-14.33**	0.72
Virginia	1.88**	-1.4**	-0.4*	-10.46**	0.82
Vermont	0.77*	-1.27**	0.23	-8.22	2.12**
Washington	-0.12	-0.81	-1.14**	0.82	-0.01
Wisconsin	1.85**	-1.04*	0.26	-5.55	0.47
West Virginia	2.58**	-0.8	-1.18**	-14.29**	3.39**
Wyoming	Reference	Reference	Reference	Reference	Reference
Year					
2013-2015	Reference	Reference	Reference	Reference	Reference
2016	0.74**	0.64**	0.03*	-0.26	-0.12*
2017	-0.12**	0.93**	0.05**	-0.4	-0.5**

© 2022 Pozniak A et al. *JAMA Health Forum.*

	Health Care Utilization During HH Episodes			Combined Spending Model [a]	
	Unplanned acute care hospitalizations among 1st HH episodes	Outpatient ED use among 1st HH episodes	Skilled Nursing Facility Use	Pre-PDGM	Post-PDGM
2018	-0.35**	0.76**	-0.06**	0.96**	-1.08**
2019	-0.42**	0.89**	-0.11**	4.79**	-1.79**
2020	-3.06**	-0.3**	-1.75**		2.71**
Intervention*2016 [b]	-0.23**	0.26**	-0.19**	-1.12**	-0.11
Intervention*2017 [c]	-0.04	0.23**	-0.2**	-2**	-0.03
Intervention*2018 [d]	-0.14	0.37**	-0.28**	-1.99**	-0.03
Intervention*2019 [e]	-0.27**	0.36**	-0.29**	-2.68**	-0.08
Intervention*2020 [f]	-0.08	0.22**	-0.6**		-3.26**
Quarter					
Quarter 1	Reference	Reference	Reference	Reference	Reference
Quarter 2	-0.24**	0.53**	-0.13**	-2.31**	0.88**
Quarter 3	-0.18**	0.64**	-0.09**	-0.73**	1.41**
Quarter 4	0.1**	0.32**	-0.06**	-1.28**	1.02**
Age					
Less than 65 years	-0.31**	1.32**	-1.1**	-5.39**	0.91**
65-84 years	Reference	Reference	Reference	Reference	Reference
85 years or older	0.67**	0.79**	0.79**	-9.52**	0.75**
Female	-1.24**	0.68**	-0.11**	-11.46**	0.43**
Race/Ethnicity (Mutually Exclusive)					
Non-Hispanic Black	0.57**	1.03**	-0.54**	8.44**	1.69**
Hispanic	-0.14**	-0.15**	-0.94**	5.69**	-0.94**
Non-Hispanic Other Race	-0.49**	-2.69**	-0.91**	1.27**	-1.14**
Non-Hispanic Multiracial	-0.42**	0.11	-0.42**	0.94	0.2
Non-Hispanic White	Reference	Reference	Reference	Reference	Reference
Dual Eligible	0.81**	2.02**	0.05**	-0.44**	1.88**
Rural	-0.31**	1.7**	0.31**	-6.92**	0.83**

© 2022 Pozniak A et al. *JAMA Health Forum.*

	Health Care Utilization During HH Episodes			Combined Spending Model [a]	
	**Unplanned acute care hospitalizations among 1st HH episodes**	**Outpatient ED use among 1st HH episodes**	**Skilled Nursing Facility Use**	**Pre-PDGM**	**Post-PDGM**
Original Reason for Medicare Entitlement					
ESRD	11.99**	0.65**	1.18**	138.34**	4.43**
Disability	1.08**	1.54**	0.2**	4.22**	1.3**
Age	Reference	Reference	Reference	Reference	Reference
Current Reason for Medicare Entitlement					
ESRD	2.99**	0.95**	-0.12**	29.89**	-0.28
Disability	0.34**	1.19**	-0.13**	9.48**	-1.14**
Age	Reference	Reference	Reference	Reference	Reference
Hospital-based HHA	0.21**	0.16	0.38**	1.28	-1.22**
Ownership					
Non-profit	0.34**	0.1	0.21**	1.99**	-2.57**
Government-owned	0.61**	0.93**	0.55**	2.17*	-1.51**
For-profit	Reference	Reference	Reference	Reference	Reference
HHA Size					
1-59 OASIS episodes	-1.99**	-1.22**	-0.58**	-15.21**	1.6**
60-249 OASIS episodes	-0.86**	-1.01**	-0.41**	-8.35**	1.9**
250-499 OASIS episodes	-0.48**	-0.44**	-0.2**	-4.53**	1.65**
500-999 OASIS episodes	-0.24**	0.03	-0.08**	-2.05**	0.82**
1,000 or more OASIS episodes	Reference	Reference	Reference	Reference	Reference
HHA Age					
0-3 years	-0.51**	-0.36**	-0.11**	1.06*	-0.05
4-10 years	-0.46**	-0.36**	-0.13**	0.17	0.17
11 years or more	Reference	Reference	Reference	Reference	Reference
HHA Chain					

© 2022 Pozniak A et al. *JAMA Health Forum.*

	Health Care Utilization During HH Episodes			Combined Spending Model [a]	
	**Unplanned acute care hospitalizations among 1st HH episodes**	**Outpatient ED use among 1st HH episodes**	**Skilled Nursing Facility Use**	**Pre-PDGM**	**Post-PDGM**
Missing	-0.36*	-0.97**	0.01	0.38	-0.75*
Undetermined	-0.001	-0.55**	0.04	2.17	-0.83**
Not Affiliated	0.03	-0.68**	-0.1**	0.43	-0.1
Affiliated	Reference	Reference	Reference	Reference	Reference
% of Persons in the Patient's County of Residence who are Ages 25 years and Older with Less than a High School Diploma	-0.003	-0.06**	-0.02**	-0.35**	0.11**
Ambulation and Locomotion					
Needs no human assistance or assistive device	Reference	Reference	Reference	Reference	Reference
Requires two handed device for level ground or human assistance for stairs and uneven ground	1.52**	-0.36**	0.93**	12.54**	0.78**
Walks only with supervision or assistance from another at all times	2.62**	-0.004	1.42**	21.99**	0.45**
Chairfast to bedfast	5.54**	-0.62**	1.34**	41.01**	3.33**
Risk for Hospitalization					
Multiple hospitalizations in past 6 months	6.57**	1.63**	1.05**	33.53**	2.71**
History of falls	1.07**	1.75**	1.08**	7.09**	1.29**

© 2022 Pozniak A et al. *JAMA Health Forum.*

	Health Care Utilization During HH Episodes			Combined Spending Model [a]	
	**Unplanned acute care hospitalizations among 1st HH episodes**	**Outpatient ED use among 1st HH episodes**	**Skilled Nursing Facility Use**	**Pre-PDGM**	**Post-PDGM**
Currently taking 5 or more medications	1.83**	1.13**	0.34**	10.35**	0.98**
Surgical wound	-2.55**	-0.7**	-1.48**	8.56**	-4.32**
Requires urinary catheter	3.54**	4.4**	-0.29**	-13.12**	2.96**
Discharged from Inpatient Facility in last 14 Days	1.59**	1.63**	0.85**	11.92**	-1.04**
Pressure Ulcer					
Stage 1	Reference	Reference	Reference	Reference	Reference
Stage 2	4.37**	-0.55**	0.68**	23.36**	1.92**
Stage 3	4**	-1.38**	0.21**	29.73**	2.57**
Stage 4	3.22**	-2.71**	-1.1**	34.66**	1.65**
Not Stageable	5.61**	-0.71**	0.85**	44.29**	3.69**
Neoplasm Diagnosis	6.54**	-0.03	0.44**	62.93**	1.05**
HIPPS Code Clinical Grouping					
Behavioral Health	-0.08	2.71**	0.05	-8.96**	1.62**
Complex Nursing Interventions	5.11**	2.74**	-0.57**	2.74**	4.63**
Surgical Aftercare	-0.54**	0.58**	-0.78**	-22.22**	-0.18*
Cardiac and Circulatory	3.63**	0.21**	0.29**	7.2**	2.18**
Endocrine	1.39**	0.23**	0.37**	7.13**	2.22**
Gastrointestinal Tract and Genitourinary System	4.47**	1.22**	0.61**	8.88**	0.7**
Infectious Disease, Neoplasms, and	6.23**	0.45**	0.2**	37.45**	2.07**

© 2022 Pozniak A et al. *JAMA Health Forum.*

	Health Care Utilization During HH Episodes			Combined Spending Model [a]	
	**Unplanned acute care hospitalizations among 1st HH episodes**	**Outpatient ED use among 1st HH episodes**	**Skilled Nursing Facility Use**	**Pre-PDGM**	**Post-PDGM**
Blood-forming Diseases					
Other	1.32**	0.99**	0.19**	1.83**	-0.3**
Respiratory	1**	-0.8**	-0.09**	-8.73**	0.35**
Musculoskeletal Rehabilitation	-4.69**	-1.7**	-0.98**	-23.28**	-3.25**
Neuro/stroke Rehabilitation	-0.88**	1.2**	-0.24**	-2.23**	-0.04
Wounds	2.71**	-0.45**	0.79**	16.06**	3.35**
None	Reference	Reference	Reference	Reference	Reference
Admission Source					
ACH	5.11**	-0.21**	-0.22**	24.72**	-3.26**
IPF	0.91**	5.12**	0.65**	18.88**	1.6**
IRF	3.88**	-0.77**	0.3**	30.69**	-0.08
LTCH	7.29**	-0.9**	-0.22**	53.95**	-0.65**
SNF	3.76**	-0.96**	2.09**	20.37**	-0.22**
Community	Reference	Reference	Reference	Reference	Reference
Oxygen Therapy	9.86**	-0.18**	1.44**	45.19**	1.2**
Alignment with selected APMs					
BPCI Model 2	-1.48**	-0.48**	0.04	-4.36**	-5.43**
BPCI Model 3	-0.15	-0.21	0.76**	6.91*	-4.37**
BPCI Advanced	0.11	-0.68**	0.32**	6.84**	-0.64*
MSSP	-0.16**	0.07**	-0.17**	0.15	-0.52**
Next Generation ACO	-0.12*	-0.28**	0.1**	-0.32	-0.26*
Pioneer ACO	-0.13	-0.03	0.2**	1.34**	0.53**
CJR	-4.01**	-0.84**	-0.94**	-34.6**	-4.26**
OCM	3.93**	0.02	0.23**	68.24**	-0.73**

© 2022 Pozniak A et al. *JAMA Health Forum.*

	Health Care Utilization During HH Episodes			Combined Spending Model [a]	
	**Unplanned acute care hospitalizations among 1st HH episodes**	**Outpatient ED use among 1st HH episodes**	**Skilled Nursing Facility Use**	**Pre-PDGM**	**Post-PDGM**
Review Choice Demonstration					
Active Participant	0.24**	0.24**	0.47**	-0.34	0.07
Inactive Participant	0.7**	0.74**	0.38**	-1.94	5.25**
Non-Participant	Reference	Reference	Reference	Reference	Reference
Intervention*Ambulation and Locomotion: Requires two handed device for level ground or human assistance for stairs and uneven ground	0.11	0.22**	0.39**	-0.35	0.09
Intervention*Ambulation and Locomotion: Walks only with supervision or assistance from another at all times	0.25**	0.17*	0.52**	-0.73	0.44**
Intervention*Ambulation and Locomotion: Chairfast to bedfast	0.32**	0.35**	0.55**	-3.62**	0.7**
Initial COVID-19 diagnosis 61-90 days before HH episode start date	1.14**	1.4**	-0.05		10.85**
Initial COVID-19 diagnosis 31-60 days before HH episode start date	-1.8**	0.34*	-0.14**		-3.11**
Initial COVID-19 diagnosis 1-30 days	-3.52**	0.75**	0.3**		39.87**

© 2022 Pozniak A et al. *JAMA Health Forum.*

	Health Care Utilization During HH Episodes			Combined Spending Model [a]	
	**Unplanned acute care hospitalizations among 1st HH episodes**	**Outpatient ED use among 1st HH episodes**	**Skilled Nursing Facility Use**	**Pre-PDGM**	**Post-PDGM**
before HH episode start date					
Initial COVID-19 diagnosis during HH episode	36.06**	2.41**	13.8**		377.44**
Initial COVID-19 diagnosis 1-30 days following HH episode end date	44.64**	2.12**	13.93**		232.58**
No COVID-19 diagnosis or initial COVID-19 diagnosis > 90 days before HH episode start date	Reference	Reference	Reference		Reference
Regional rates of Medicare FFS inpatient stays associated with COVID-19 diagnoses	-0.01**	-0.02**	-0.0004		-0.14**
County-level rates of COVID-19 Diagnoses					
Greater than or equal to 75th Percentile	-0.66**	-0.56**	-0.81**		-4.05**
25th to 75th Percentile	-0.09	-1.05**	-0.68**		-7.52**
Less than 25th Percentile	Reference	Reference	Reference		Reference
State*Linear time trend					
Alaska*Linear time trend				3.6**	0.67
Alabama*Linear time trend				1.05**	0.22*

© 2022 Pozniak A et al. *JAMA Health Forum.*

	Health Care Utilization During HH Episodes			Combined Spending Model [a]	
	Unplanned acute care hospitalizations among 1st HH episodes	Outpatient ED use among 1st HH episodes	Skilled Nursing Facility Use	Pre-PDGM	Post-PDGM
Arkansas*Linear time trend				2.09**	-0.04
Arizona*Linear time trend				0.86**	0.65**
California*Linear time trend				2.07**	0.56**
Colorado*Linear time trend				2.7**	-0.06
Connecticut*Linear time trend				1.65**	0.25
Delaware*Linear time trend				1.53**	0.68**
Florida*Linear time trend				1.27**	0.3**
Georgia*Linear time trend				1.79**	0.41**
Hawaii*Linear time trend				3.42**	-0.35
Iowa*Linear time trend				2.99**	0.69**
Idaho*Linear time trend				1.07**	0.55**
Illinois*Linear time trend				2.81**	-0.13*
Indiana*Linear time trend				1.48**	0.1
Kansas*Linear time trend				1.64**	0.54**
Kentucky*Linear time trend				1.36**	0.12

© 2022 Pozniak A et al. *JAMA Health Forum.*

	Health Care Utilization During HH Episodes			Combined Spending Model [a]	
	Unplanned acute care hospitalizations among 1st HH episodes	Outpatient ED use among 1st HH episodes	Skilled Nursing Facility Use	Pre-PDGM	Post-PDGM
Louisiana*Linear time trend				1.26**	0.34**
Massachusetts*Linear time trend				2.54**	0.14
Maryland*Linear time trend				1.53**	0.88**
Maine*Linear time trend				2.6**	0.15
Michigan*Linear time trend				1.6**	0.14
Minnesota*Linear time trend				3.24**	0.42*
Missouri*Linear time trend				1.93**	0.56**
Mississippi*Linear time trend				2.14**	0.04
Montana*Linear time trend				2**	0.66*
North Carolina*Linear time trend				1.72**	0.59**
North Dakota*Linear time trend				2.23**	1.07*
Nebraska*Linear time trend				3.77**	0.13
New Hampshire *Linear time trend				1.87**	0.4
New Jersey*Linear time trend				4.28**	-0.34**
New Mexico*Linear time trend				1.43**	0.09

© 2022 Pozniak A et al. *JAMA Health Forum.*

	Health Care Utilization During HH Episodes			Combined Spending Model [a]	
	**Unplanned acute care hospitalizations among 1st HH episodes**	**Outpatient ED use among 1st HH episodes**	**Skilled Nursing Facility Use**	**Pre-PDGM**	**Post-PDGM**
Nevada*Linear time trend				2.56**	0.01
New York*Linear time trend				1.83**	0.5**
Ohio*Linear time trend				1.8**	0.25**
Oklahoma*Linear time trend				1.78**	0.29**
Oregon*Linear time trend				2.59**	0.51**
Pennsylvania*Linear time trend				1.89**	0.17*
Rhode Island*Linear time trend				2.15**	-0.26
South Carolina*Linear time trend				0.8**	0.7**
South Dakota*Linear time trend				2.99**	0.09
Tennessee*Linear time trend				1.92**	-0.08
Texas*Linear time trend				2.44**	0.34**
Utah*Linear time trend				1.93**	0.54**
Virginia*Linear time trend				1.45**	0.26**
Vermont*Linear time trend				-1.78	0.6**
Washington*Linear time trend				1.76**	0.13

© 2022 Pozniak A et al. *JAMA Health Forum.*

	**Health Care Utilization During HH Episodes**			**Combined Spending Model [a]**	
	**Unplanned acute care hospitalizations among 1st HH episodes**	**Outpatient ED use among 1st HH episodes**	**Skilled Nursing Facility Use**	**Pre-PDGM**	**Post-PDGM**
Wisconsin*Linear time trend				1.75**	0.55**
West Virginia*Linear time trend				1.46**	0.21
Wyoming*Linear time trend				2.43**	0.31

Abbreviations: ACH, acute care hospitalization; ACO, accountable care organization; APM, alternative payment model; BPCI, bundled payment for care improvement; CJR, comprehensive care for joint replacement; D-in-D, difference-in-differences; ED, emergency department; ESRD, end-stage renal disease; FFS, fee-for-service; HHA, home health agency; HH, home health; HIPPS, health insurance prospective payment system; IPF, inpatient psychiatric facility; IRF, inpatient rehabilitation facility; LTCH, long-term care hospital; MSSP, Medicare shared savings program; OASIS, Outcome and Assessment Information Set; OCM, oncology care model; PDGM, Patient Driven Groupings Model; SNF, skilled nursing facility.

* P<.10; ** P<.05. [a] Parameter estimates for 2020 for spending measure is the sum of pre-PDGM and post-PDGM regression coefficients.

[b] D-in-D estimate for 2016. [c] D-in-D estimate for 2017. [d] D-in-D estimate for 2018. [e] D-in-D estimate for 2019. [f] D-in-D estimate for 2020.

© 2022 Pozniak A et al. *JAMA Health Forum.*

eTable 6. Multivariate D-in-D Regression Results for the OASIS-based Measures

	OASIS-based Quality Measures					
	**Discharged to community**	**TNC change in self-care**	**TNC change in mobility**	**Improvement in dyspnea**	**Improvement in management of oral medications**	**Improvement in pain interfering with activity**
N of OASIS-based episodes	53,570,641	39,267,062	39,267,062	29,737,485	32,664,331	33,134,830
Intercept	92.45**	0.49**	0.15**	55.72**	42.07**	49.29**
State						
Alaska	1.53	-0.3**	-0.1**	-2.71	-11.00**	-6.02**
Alabama	-6.27**	0.22**	0.04**	7.56**	10.39**	9.22**
Arkansas	-5.75**	0.16**	0.04**	3.6	8.18**	6.41**
Arizona	0.76	0.002	-0.03	6.75**	1.26	2.95
California	2.58*	0.04	-0.01	11.08**	3.55**	10.16**
Colorado	1.34	0.05	0.02	5.26**	2.8	4.68*
Connecticut	-8.23**	-0.1*	-0.03**	2.64	0.01	3.82
Delaware	1.18	0.05	0.01	6.29**	4.75*	8**
Florida	-1.87	0.17**	0.04**	3.95*	2.97	10.94**
Georgia	-1.51	0.09*	0.01	6.17**	2.78	6.29**
Hawaii	2.83*	-0.15**	-0.05**	7.91**	-6.4**	4.74*
Iowa	-7.22**	0.03	0.01	3.33	7.22**	8.07**
Idaho	0.83	0.06	0.03*	8.47**	3.93	4.5*
Illinois	-1.41	0.12**	0.02	7.71**	9.29**	11.08**
Indiana	-4.62**	0.05	0.01	4.91**	4.48**	6.28**
Kansas	-2.11	0.11**	0.03**	4.85**	4.87**	6.61**
Kentucky	-5.23**	0.13**	0.02*	5.82**	4.61**	6.97**
Louisiana	-10.73**	0.07	-0.002	-0.13	7.25**	3.33
Massachusetts	-8.14**	0.04	0.01	4.61*	7.17**	8.67**
Maryland	-0.76	0.09*	0.01	10.15**	5.51**	8.89**
Maine	-0.8	0.06	0.02	3.22	4.93**	8.39**
Michigan	1.22	0.17**	0.03**	8.19**	8.07**	8.54**
Minnesota	-3.65**	-0.13**	-0.04**	0.71	-3.73	1.78
Missouri	-0.04	0.13**	0.03**	5.84**	5.41**	5.48**

© 2022 Pozniak A et al. *JAMA Health Forum.*

	OASIS-based Quality Measures					
	**Discharged to community**	**TNC change in self-care**	**TNC change in mobility**	**Improvement in dyspnea**	**Improvement in management of oral medications**	**Improvement in pain interfering with activity**
Mississippi	-10.01**	0.16**	0.03*	3.82	10.88**	7.08**
Montana	4.07**	-0.06	0.001	3.32	-0.21	5.73*
North Carolina	-2.06	-0.03	-0.02	4.88**	-2.37	5.63**
North Dakota	-0.86	0.1*	0.03	9.49**	9.24**	10.53**
Nebraska	-2.98*	-0.01	-0.01	4.61*	0.37	8.06**
New Hampshire	-2.99*	0.01	0.01	3.92	5.33**	6.31**
New Jersey	1.05	0.12**	0.03*	10.52**	9.48**	11.14**
New Mexico	-3.25**	0.02	-0.002	4.23*	5.6**	-0.09
Nevada	-1.81	0.12**	0.02	6.68**	9.8**	4.94*
New York	-1.16	0.04	0.0005	7.77**	5.83**	8.76**
Ohio	-4.86**	0.04	0.02	3.46	3.69	3.59
Oklahoma	-13.73**	0.06	0.01	-1.86	8.15**	0.82
Oregon	0.52	-0.2**	-0.08**	0.49	-10.13**	0.01
Pennsylvania	-1.76	0.06	0.01	7.06**	4.75**	8.2**
Rhode Island	-1.89	0.13**	0.04**	8.48**	6.4**	7.85**
South Carolina	-0.31	0.18**	0.05**	9.72**	9.39**	9.02**
South Dakota	3.54*	0.05	0.02	7.76**	0.63	10.42**
Tennessee	-6.87**	0.05	-0.002	1.93	3.24	3.93
Texas	-10.12**	-0.06	-0.04**	-5.78**	0.41	-3.52
Utah	1.56	0.12**	0.04**	7.95**	6.79**	2.76
Virginia	-0.84	0.08	0.02	7.33**	3.74**	6.38**
Vermont	-3.48**	-0.02	0.0002	1.05	3.87	6.39*
Washington	-0.67	-0.13**	-0.06**	1.13	-6.19**	0.69
Wisconsin	-0.64	-0.03	-0.01	4.69*	0.19	6.18**
West Virginia	-4.95**	0.17**	0.05**	6.45**	9.09**	7.41**
Wyoming	Reference	Reference	Reference	Reference	Reference	Reference
Year						
2013-2015	Reference	Reference	Reference	Reference	Reference	Reference
2016	0.34**	0.05**	0.02**	1.37**	1.83**	2.69**
2017	0.73**	0.08**	0.04**	2.91**	3.56**	4.79**

© 2022 Pozniak A et al. *JAMA Health Forum.*

	OASIS-based Quality Measures					
	**Discharged to community**	**TNC change in self-care**	**TNC change in mobility**	**Improvement in dyspnea**	**Improvement in management of oral medications**	**Improvement in pain interfering with activity**
2018	1.17**	0.09**	0.05**	3.22**	4.75**	6.06**
2019	1.81**	0.14**	0.07**	4.16**	8.11**	7.7**
2020	3.5**	0.2**	0.08**	4.36**	10.22**	8.4949**
Intervention*2016 [a]	0.42**	0.02**	0.01**	0.8*	1.91**	1.24**
Intervention*2017 [b]	0.55**	0.04**	0.01**	0.75	3.06**	1.75**
Intervention*2018 [c]	0.97**	0.05**	0.02**	0.06	3.19**	1.97**
Intervention*2019 [d]	1.19**	0.05**	0.02*	-0.4	2.61**	2.48**
Intervention*2020 [e]	1.39**	0.04	0.01	-1.49	1.58	2.58**
Quarter						
Quarter 1	Reference	Reference	Reference	Reference	Reference	Reference
Quarter 2	0.96**	0.02**	0.01**	0.59**	0.95**	0.61**
Quarter 3	0.91**	0.03**	0.01**	0.85**	1.64**	1.05**
Quarter 4	1.33**	0.04**	0.02**	1.17**	2.42**	1.45**
Age						
Less than 65 years of age	-2.56**	0.03**	0.01**	-0.39**	3.76**	-5.55**
65-84 years of age	Reference	Reference	Reference	Reference	Reference	Reference
85 years of age or older	-1.13**	-0.25**	-0.1**	-1.68**	-12.54**	2.31**
Female	2.14**	0.03**	-0.01**	0.85**	2.17**	-2.21**
Race/Ethnicity (Mutually Exclusive)						
Non-Hispanic Black	-0.04	-0.05**	-0.02**	0.96**	-0.11	1.39**
Hispanic	2.17**	-0.08**	-0.02**	-1.5**	-6.13**	1.89**
Non-Hispanic Other Race	3.67**	-0.04**	0.001	1.81**	-2.56**	2.13**
Non-Hispanic Multiracial	-0.17	-0.03**	-0.01**	0.01	-0.91**	-1.25**
Non-Hispanic White	Reference	Reference	Reference	Reference	Reference	Reference
Dual Eligible	-4.78**	-0.12**	-0.04**	-3.68**	-4.02**	-1.64**

© 2022 Pozniak A et al. *JAMA Health Forum.*

	OASIS-based Quality Measures					
	**Discharged to community**	**TNC change in self-care**	**TNC change in mobility**	**Improvement in dyspnea**	**Improvement in management of oral medications**	**Improvement in pain interfering with activity**
Medicaid	-2.37**	-0.13**	-0.04**	-2.2**	-2.48**	-2.45**
Rural	-2.45**	0.05**	0.02**	-0.03	2.14**	-1.12**
Hospital-based HHA	0.9**	-0.05**	-0.02**	-0.3	-1.82**	-1.82**
Ownership						
Non-profit	2.98**	0.03**	0.001	0.18	2.3**	-2.29**
Government-owned	2.2**	-0.02	-0.01	-0.02	-0.52	-2.92**
For-profit	Reference	Reference	Reference	Reference	Reference	Reference
HHA Size						
1-59 OASIS episodes	-9.63**	-0.36**	-0.12**	-16.97**	-13.69**	-13.82**
60-249 OASIS episodes	-5.01**	-0.16**	-0.07**	-8.77**	-7.62**	-4.19**
250-499 OASIS episodes	-2.31**	-0.07**	-0.04**	-4.37**	-4.16**	-0.96**
500-999 OASIS episodes	-0.97**	-0.02**	-0.01**	-1.62**	-1.78**	-0.25
1,000 or more OASIS episodes	Reference	Reference	Reference	Reference	Reference	Reference
HHA Age						
0-3 years	2.55**	0.05**	0.01**	0.3	-0.12	2.65**
4-10 years	0.64**	0.01	-0.001	-0.49*	-0.96**	1.46**
11 years or more	Reference	Reference	Reference	Reference	Reference	Reference
HHA Chain						
Missing	2.05**	-0.02	-0.01	-5.05**	-2.5**	0.17
Undetermined	1.3**	-0.01	0.001	-1.16*	0.11	-0.11
Not Affiliated	0.44**	-0.05**	-0.03**	-3.25**	-2.84**	-1.37**
Affiliated	Reference	Reference	Reference	Reference	Reference	Reference
% of Persons in the Patient's County of Residence who are Ages 25 years and	-0.1**	-0.003**	-0.001**	-0.21**	0.04	0.07**

© 2022 Pozniak A et al. *JAMA Health Forum.*

	OASIS-based Quality Measures					
	**Discharged to community**	**TNC change in self-care**	**TNC change in mobility**	**Improvement in dyspnea**	**Improvement in management of oral medications**	**Improvement in pain interfering with activity**
Older with Less than a High School Diploma						
Ambulation and Locomotion						
Needs no human assistance or assistive device	Reference	Reference	Reference	Reference	Reference	Reference
Requires two handed device for level ground or human assistance for stairs and uneven ground	-2.5**	0.14**	0.03**	0.41**	-0.91**	-2.09**
Walks only with supervision or assistance from another at all times	-3.64**	0.26**	0.16**	4.58**	1.96**	1.73**
Chairfast to bedfast	-13.88**	-0.41**	-0.13**	-3.55**	-19.12**	-4.19**
Risk for Hospitalization						
Multiple hospitalizations in past 6 months	-14.86**	-0.1**	-0.04**	-4.62**	-2.25**	-2.71**
History of falls	-0.17**	-0.02**	-0.02**	0.73**	-3.14**	-1.26**
Currently taking 5 or more medications	-4.73**	0.03**	-0.002	-2.64**	0.32**	-1.37**
Surgical wound	9.55**	0.38**	0.12**	9.41**	14.69**	3.65**
Requires urinary catheter	-14.21**	-0.21**	-0.11**	-2.74**	-4.32**	-1.8**
Discharged from Inpatient Facility in last 14 Days	-7.4**	0.21**	0.07**	2.77**	8.43**	1.34**
Pressure Ulcer						

© 2022 Pozniak A et al. *JAMA Health Forum.*

	OASIS-based Quality Measures					
	**Discharged to community**	**TNC change in self-care**	**TNC change in mobility**	**Improvement in dyspnea**	**Improvement in management of oral medications**	**Improvement in pain interfering with activity**
Stage 1	Reference	Reference	Reference	Reference	Reference	Reference
Stage 2	-11.32**	-0.38**	-0.18**	-6.11**	-9.18**	-3.68**
Stage 3	-13.09**	-0.32**	-0.17**	-4.84**	-5.61**	-4.04**
Stage 4	-15.02**	-0.41**	-0.25**	-5.69**	-6.76**	-8.13**
Not Stageable	-14.55**	-0.34**	-0.16**	-5.51**	-9**	-4.75**
Neoplasm Diagnosis	-13.61**	-0.14**	-0.04**	-6.44**	-2.81**	-3.47**
Intervention*Ambulation and Locomotion: Requires two handed device for level ground or human assistance for stairs and uneven ground	2.34**	0.02**	0.01	1.18**	2.6**	0.96**
Intervention*Ambulation and Locomotion: Walks only with supervision or assistance from another at all times	2.35**	0.01	0.002	1.66**	3.81**	0.53
Intervention*Ambulation and Locomotion: Chairfast to bedfast	2.13**	-0.01	0.01	2.09**	4.01**	1.07**
Regional rates of Medicare FFS inpatient stays associated with COVID-19 diagnoses	0.01**	-0.001**	-0.0004**	-0.01*	-0.02**	0.004
County-level rates of COVID-19 Diagnoses						
Greater than or equal to 75th Percentile	-1.23**	0.03**	0.01*	-1.14**	-0.4*	-1.74**
25th to 75th Percentile	-1.26**	0.01*	-0.001	-0.96**	-0.49**	-0.9**

© 2022 Pozniak A et al. *JAMA Health Forum.*

	OASIS-based Quality Measures					
	**Discharged to community**	**TNC change in self-care**	**TNC change in mobility**	**Improvement in dyspnea**	**Improvement in management of oral medications**	**Improvement in pain interfering with activity**
Less than 25th Percentile	Reference	Reference	Reference	Reference	Reference	Reference
State*Linear time trend						
Alaska*Linear time trend	-0.46	0.02*	0.004	0.57*	1.63**	0.05
Alabama*Linear time trend	-0.03	0.04**	0.02**	2.05**	2.44**	1.79**
Arkansas*Linear time trend	0.31**	0.04**	0.02**	2.36**	2.04**	1.84**
Arizona*Linear time trend	-0.55**	0.03**	0.01**	1.54**	1.98**	1.18**
California*Linear time trend	-0.19**	0.02**	0.005**	1.17**	1.57**	0.6**
Colorado*Linear time trend	-0.15	0.04**	0.01**	1.69**	1.86**	1.37**
Connecticut*Linear time trend	0.32**	0.04**	0.01**	1.97**	1.94**	1.35**
Delaware*Linear time trend	-0.39*	0.05**	0.02**	1.69**	1.94**	1.3**
Florida*Linear time trend	-0.39**	0.01**	0.01**	2.2**	2.27**	0.06
Georgia*Linear time trend	-0.37**	0.04**	0.02**	2.08**	2.57**	1.76**
Hawaii*Linear time trend	-0.24	0.03**	0.01	1.09**	2.02**	0.73**
Iowa*Linear time trend	-0.18	0.02**	0.001	0.91**	0.34	-0.39*
Idaho*Linear time trend	-0.26*	0.02**	0.01**	1.16**	1.72**	1.22**
Illinois*Linear time trend	0.17**	0.02**	0.01**	1.19**	0.88**	0.27*

© 2022 Pozniak A et al. *JAMA Health Forum.*

	OASIS-based Quality Measures					
	**Discharged to community**	**TNC change in self-care**	**TNC change in mobility**	**Improvement in dyspnea**	**Improvement in management of oral medications**	**Improvement in pain interfering with activity**
Indiana*Linear time trend	0.36**	0.04**	0.01**	1.62**	1.96**	0.96**
Kansas*Linear time trend	-0.1	0.03**	0.01**	1.57**	1.88**	0.75**
Kentucky*Linear time trend	0.25	0.04**	0.01**	1.77**	2.31**	1.36**
Louisiana*Linear time trend	1.11**	0.05**	0.02**	3.24**	2.65**	2.35**
Massachusetts*Linear time trend	-0.53**	0.03**	0.01**	1.17**	0.63**	-0.18
Maryland*Linear time trend	-0.33*	0.04**	0.02**	1.16**	1.57**	0.94**
Maine*Linear time trend	-0.14	0.03**	0.01**	0.85**	1.17**	0.19
Michigan*Linear time trend	-0.02	0.02**	0.01**	1.06**	1.34**	0.62**
Minnesota*Linear time trend	0.12	0.02**	0.005	0.76**	0.77	0.63*
Missouri*Linear time trend	0.03	0.03**	0.01**	1.46**	1.8**	0.69**
Mississippi*Linear time trend	0.88**	0.04**	0.02**	2.57**	2.11**	2.07**
Montana*Linear time trend	-0.38*	0.03*	0.001	0.5	0.56	-0.62
North Carolina*Linear time trend	-0.55**	0.04**	0.02**	1.74**	2.84**	1.06**
North Dakota*Linear time trend	-0.39	0.02	0.004	1.05**	0.8	0.67
Nebraska*Linear time trend	-0.51**	0.03**	0.01**	1.24**	1.46**	0.11

© 2022 Pozniak A et al. *JAMA Health Forum.*

	OASIS-based Quality Measures					
	**Discharged to community**	**TNC change in self-care**	**TNC change in mobility**	**Improvement in dyspnea**	**Improvement in management of oral medications**	**Improvement in pain interfering with activity**
New Hampshire *Linear time trend	0.05	0.05**	0.02**	1.55**	1.78**	1.19**
New Jersey*Linear time trend	-0.33**	0.04**	0.01**	1.27**	1.05**	0.9**
New Mexico*Linear time trend	0.01	0.02**	0.004	1.06**	0.99**	0.63**
Nevada*Linear time trend	-0.39*	0.01*	0.004	0.74**	0.45	0.54
New York*Linear time trend	0.48**	0.05**	0.02**	2**	1.63**	1.12**
Ohio*Linear time trend	0.22	0.04**	0.01**	1.53**	2.18**	1.07**
Oklahoma*Linear time trend	0.46**	0.04**	0.01**	2.18**	1.69**	1.66**
Oregon*Linear time trend	0.09	0.03**	0.01**	0.97**	1.74**	0.76**
Pennsylvania*Linear time trend	-0.3**	0.04**	0.01**	1.38**	1.71**	0.84**
Rhode Island*Linear time trend	-0.24*	0.06**	0.02**	1.23**	1.25**	0.96**
South Carolina*Linear time trend	-0.35**	0.04**	0.02**	1.01**	1.48**	0.71**
South Dakota*Linear time trend	-0.04	0.03**	0.01**	0.78**	2.08**	-0.14
Tennessee*Linear time trend	-0.25*	0.04**	0.01**	2.69**	2.82**	2.07**
Texas*Linear time trend	0.65**	0.04**	0.01**	2.76**	2.46**	2.06**
Utah*Linear time trend	-0.06	0.03**	0.01**	0.69**	1.28**	1.63**
Virginia*Linear time trend	-0.14	0.04**	0.01**	1.51**	1.85**	1**

© 2022 Pozniak A et al. *JAMA Health Forum.*

	OASIS-based Quality Measures					
	**Discharged to community**	**TNC change in self-care**	**TNC change in mobility**	**Improvement in dyspnea**	**Improvement in management of oral medications**	**Improvement in pain interfering with activity**
Vermont*Linear time trend	-0.16	0.03**	0.01**	1.08**	1.13**	0.69**
Washington*Linear time trend	-0.25*	0.06**	0.02**	2.07**	2.64**	1.6**
Wisconsin*Linear time trend	0.1	0.04**	0.01**	1.52**	1.82**	1.07**
West Virginia*Linear time trend	-0.19	0.06**	0.02**	2.28**	2.49**	2.25**
Wyoming*Linear time trend	-0.03	0.03**	0.01**	0.96**	1.59**	-0.25
Start of Care TNC Self-Care: Less than 8 OASIS responses		Reference				
Start of Care TNC Self-Care: 8-10 OASIS responses		0.66**				
Start of Care TNC Self-Care: 11-15 OASIS responses		1.05**				
Start of Care TNC Self-Care: 16 or more OASIS responses		1.16**				
Intervention*Start of Care TNC Self-Care: 8-10 OASIS responses		0.04**				
Intervention*Start of Care TNC Self-Care: 11-15 OASIS responses		0.04**				
Intervention*Start of Care TNC Self-Care: 16		0.07**				

© 2022 Pozniak A et al. *JAMA Health Forum.*

	OASIS-based Quality Measures					
	**Discharged to community**	**TNC change in self-care**	**TNC change in mobility**	**Improvement in dyspnea**	**Improvement in management of oral medications**	**Improvement in pain interfering with activity**
or more OASIS responses						
Start of Care TNC Mobility: Less than 4 OASIS responses			Reference			
Start of Care TNC Mobility: 4-6 OASIS responses			0.26**			
Start of Care TNC Mobility: 7-9 OASIS responses			0.53**			
Start of Care TNC Mobility: 10 or more OASIS responses			0.71**			
Intervention*Start of Care TNC Mobility: 4-6 OASIS responses			0.01**			
Intervention*Start of Care TNC Mobility: 7-9 OASIS responses			0.01			
Intervention*Start of Care TNC Mobility: 10 or more OASIS responses			0.004			
Start of Care Dyspnea: Patient is short of breath only when walking more than 20 feet				Reference		
Start of Care Dyspnea: Patient is dyspneic or				17.37**		

© 2022 Pozniak A et al. *JAMA Health Forum.*

	OASIS-based Quality Measures					
	**Discharged to community**	**TNC change in self-care**	**TNC change in mobility**	**Improvement in dyspnea**	**Improvement in management of oral medications**	**Improvement in pain interfering with activity**
noticeable short of breath with moderate exertion						
Start of Care Dyspnea: Start of Care Dyspnea: Patient is dyspneic or noticeable short of breath with minimal exertion or at rest				23.64**		
Intervention*Start of Care Dyspnea: Patient is dyspneic or noticeable short of breath with moderate exertion				0.09		
Intervention*Start of Care Dyspnea: Start of Care Dyspnea: Patient is dyspneic or noticeable short of breath with minimal exertion or at rest				0.3		
Start of Care Oral Medication Management: Patient is able to take oral medications if prepared in advance/another person develops a drug diary					Reference	

© 2022 Pozniak A et al. *JAMA Health Forum.*

	OASIS-based Quality Measures					
	**Discharged to community**	**TNC change in self-care**	**TNC change in mobility**	**Improvement in dyspnea**	**Improvement in management of oral medications**	**Improvement in pain interfering with activity**
Start of Care Oral Medication Management: Patient is able to take medications at the correct time if given reminders by other person at appropriate times					16.52**	
Start of Care Oral Medication Management: Patient is unable to take medication unless administered by another person					9.44**	
Intervention*Oral Medication Management: Patient is able to take medications at the correct time if given reminders by other person at appropriate times					-3.42**	
Intervention*Oral Medication Management: Patient is unable to take medication unless administered by another person					-3.56**	

© 2022 Pozniak A et al. *JAMA Health Forum.*

	OASIS-based Quality Measures					
	**Discharged to community**	**TNC change in self-care**	**TNC change in mobility**	**Improvement in dyspnea**	**Improvement in management of oral medications**	**Improvement in pain interfering with activity**
Start of Care Pain: Pain does not interfere with activity						Reference
Start of Care Pain: Pain Interferes less often with patient's activity or movement than daily pain						16.04**
Start of Care Pain: Pain Interferes daily with patient's activity or movement, but not constant pain						16.53**
Start of Care Pain: Patient is in constant pain						29.27**
Intervention*Pain: Pain Interferes less often with patient's activity or movement than daily pain						0.17*
Intervention*Pain: Pain Interferes daily with patient's activity or movement, but not constant pain						-0.66*
Intervention*Pain: Patient is in constant pain						-2.15**

Abbreviations: D-in-D, difference-in-differences; FFS, fee-for-service; HHA, home health agency; OASIS, Outcome and Assessment Information Set; TNC, total normalized composite.

© 2022 Pozniak A et al. *JAMA Health Forum.*

* P<.10; ** P<.05. [a] D-in-D estimate for 2016. [b] D-in-D estimate for 2017. [c] D-in-D estimate for 2018. [d] D-in-D estimate for 2019. [e] D-in-D estimate for 2020.

© 2022 Pozniak A et al. *JAMA Health Forum.*

eTable 7. Multivariate D-in-D Regression Results for the HHCAHPS-based Measures

	Patient Experience				
	**Professional Care**	**Communication**	**Discussion of Care**	**Overall Care**	**Likely to Recommend**
N of HHA-years	62,148	62,148	62,148	62,148	62,148
Intercept	85.17**	83.57**	77.4**	77.59**	75.25**
State					
Alaska	0.91	0.32	0.71	0.46	0.07
Alabama	4.41**	4.99**	6.62**	7.14**	6.96**
Arkansas	2.17**	2.22**	3.16**	3.69**	2.44**
Arizona	0.47	0.01	0.97	0.13	-1.95
California	-0.29	-0.51	1.28*	-0.33	-2.81**
Colorado	0.52	0.36	1.57**	0.81	-1.32
Connecticut	-0.53	-0.37	1.68**	0.14	-1.45
Delaware	1.17	1.42	3.48**	2.62	0.46
Florida	1.7**	1.5**	1.38**	2.91**	0.85
Georgia	2.28**	2.69**	2.96**	4.21**	3.42**
Hawaii	7.07**	6.86**	4.17**	8.89**	9.22**
Iowa	-0.33	-0.08	2.98**	0.76	-2.09*
Idaho	1.25*	0.58	0.25	1.61	1.18
Illinois	1.6**	1.69**	3.35**	2.68**	-0.08
Indiana	0.48	0.9	1.59**	2.04**	-1.04
Kansas	1.01	1.03	3.25**	2.15**	0.81
Kentucky	3.15**	2.94**	2.76**	5.21**	4.0**
Louisiana	4.76**	5.35**	6.91**	7.67**	7.58**
Massachusetts	1.38**	1.03	4.46**	2.49**	1.83
Maryland	1.25	1.64*	2.17**	1.93	-0.22
Maine	0.99	1.51*	1.64**	2.6**	2.62*
Michigan	1.28**	1.34*	3.0**	2.42**	-0.91
Minnesota	-1.44**	-1.63**	0.04	-1.9*	-5.05**
Missouri	1.61**	2.06**	3.88**	2.75**	0.42
Mississippi	4.91**	5.28**	6.52**	7.8**	7.99**
Montana	-0.42	-0.71	-0.44	-0.58	-2.77*
North Carolina	3.54**	3.77**	4.5**	5.67**	4.07**

© 2022 Pozniak A et al. *JAMA Health Forum.*

	Patient Experience				
	**Professional Care**	**Communication**	**Discussion of Care**	**Overall Care**	**Likely to Recommend**
North Dakota	0.57	0.44	3.76**	0.89	-0.36
Nebraska	-0.33	0.31	1.69*	-0.15	-2.38*
New Hampshire	0.83	0.85	2.55**	2.43**	2.85*
New Jersey	0.17	0.56	3.04**	0.79	-1.28
New Mexico	1.76**	1.45*	3.11**	2.68**	1.65
Nevada	1.07	-0.59	2.11**	-0.18	-0.01
New York	-3.12**	-2.7**	-0.33	-3.72**	-6.7**
Ohio	0.45	0.3	1.91**	1.38	-2.8**
Oklahoma	2.22**	2.27**	4.26**	3.55**	2.07*
Oregon	-0.91	-1.43*	-0.97	-2.6**	-3.53**
Pennsylvania	2.32**	2.84**	3.63**	4.4**	1.34
Rhode Island	1.3*	2.07**	4.09**	2.72**	1.37
South Carolina	4.01**	4.6**	5.29**	6.75**	6.29**
South Dakota	1.41*	0.91	3.22**	2.08*	0.77
Tennessee	3.04**	3.33**	3.69**	4.49**	3.76**
Texas	2.22**	2.24**	5.43**	4.11**	2.73**
Utah	0.13	-1.41	-0.72	-0.18	-0.95
Virginia	1.36**	1.65**	1.25*	2.53**	0.81
Vermont	-0.61	-0.78	0.27	-0.42	1.85
Washington	-0.43	-1.12	0.02	-1.29	-2.76**
Wisconsin	-0.25	-0.04	1.24	-0.11	-2.77**
West Virginia	3.25**	3.6**	3.19**	5.35**	4.88**
Wyoming	Reference	Reference	Reference	Reference	Reference
Year					
2013-2015	Reference	Reference	Reference	Reference	Reference
2016	-0.08	-0.04	-0.03	0.21*	-0.17
2017	-0.18**	-0.14*	-0.25**	-0.04	-0.65**
2018	-0.01	0.07	-0.05	0.02	-0.72**
2019	0.09	0.07	0.09	0.37**	-0.53**
2020	1.53	0.68	0.81	1.09	-3.38
Intervention*2016 [a]	-0.11	-0.22	-0.35*	-0.1	0.01
Intervention*2017 [b]	0.02	-0.05	0.22	0.03	0.29

© 2022 Pozniak A et al. *JAMA Health Forum.*

	Patient Experience				
	**Professional Care**	**Communication**	**Discussion of Care**	**Overall Care**	**Likely to Recommend**
Intervention*2018 [c]	-0.08	-0.3*	-0.24	0.25	0.39
Intervention*2019 [d]	-0.41**	-0.41**	-0.62**	-0.18	-0.03
Intervention*2020 [e]	-0.48**	-0.22	-0.65**	-0.23	-0.72**
Female	2.3**	1.03	1.67**	5.18**	4.22**
Race/Ethnicity (Mutually Exclusive)					
Non-Hispanic Black	-3.55**	-6.23**	-3.47**	-7.49**	-10.94**
Hispanic	-0.37	-1.46**	-4.93**	-0.96**	-2.38**
Non-Hispanic Other Race	-10.56**	-9.05**	-2.97**	-12.72**	-13.89**
Non-Hispanic Multiracial	3.51	-2.56	-4.23	1.04	1.22
Non-Hispanic White	Reference	Reference	Reference	Reference	Reference
Dual Eligible	1.76**	2.56**	9.19**	3.27**	4.48**
Medicaid	-4.46**	-4.7**	-5.25**	-5.07**	-8.23**
Rural	1.98**	1.92**	2.17**	2.91**	4.56**
Hospital-based HHA	1.04**	1.36**	2.13**	1.38**	1.52**
Ownership					
Non-profit	0.96**	1.06**	1.48**	1.73**	2.93**
Government-owned	1.46**	1.42**	1.69**	2.74**	4.14**
For-profit	Reference	Reference	Reference	Reference	Reference
HHA Size					
1-59 OASIS episodes	-0.27	-0.44*	0.7**	-0.98**	-1.84**
60-249 OASIS episodes	0.24**	0.14	0.69**	0.18	-0.25
250-499 OASIS episodes	0.01	-0.03	-0.22*	0.004	-0.41**
500-999 OASIS episodes	-0.05	0.02	-0.22*	-0.13	-0.37**
1,000 or more OASIS episodes	Reference	Reference	Reference	Reference	Reference

© 2022 Pozniak A et al. *JAMA Health Forum.*

	Patient Experience				
	**Professional Care**	**Communication**	**Discussion of Care**	**Overall Care**	**Likely to Recommend**
HHA Age					
0-3 years	-0.36**	-0.64**	-0.29	-1.11**	-0.72**
4-10 years	-0.35**	-0.69**	-0.32**	-0.84**	-0.89**
11 years or more	Reference	Reference	Reference	Reference	Reference
HHA Chain					
Missing	-0.31	-0.38	-0.55*	0.03	-0.62
Undetermined	0.20	0.05	-0.26	0.55**	0.47
Not Affiliated	0.48**	0.22**5	-0.7**	0.95**	1.03**
Affiliated	Reference	Reference	Reference	Reference	Reference
Regional rates of Medicare FFS inpatient stays associated with COVID-19 diagnoses	0.01	0.02	0.04**	0.03	0.02
County-level rates of COVID-19 Diagnoses					
Greater than or equal to 75th Percentile	-1.71	-1.04	-3.73*	-0.19	3.11
25th to 75th Percentile	-1.71	-1.09	-2.51	-1.28	3.9
Less than 25th Percentile	Reference	Reference	Reference	Reference	Reference

Abbreviations: D-in-D, difference-in-differences; FFS, fee-for-service; HHA, home health agency; HHCAHPS, Home Health Consumer Assessment of Healthcare Providers and Systems; OASIS, Outcome and Assessment Information Set.

* P<.10; ** P<.05. [a] D-in-D estimate for 2016. [b] D-in-D estimate for 2017. [c] D-in-D estimate for 2018. [d] D-in-D estimate for 2019. [e] D-in-D estimate for 2020.

© 2022 Pozniak A et al. *JAMA Health Forum.*

eTable 8. Sample Sizes for Analyses of Utilization, Medicare Payments, OASIS-based Quality, and HHCAHPS-based Measures

Measure	HHVBP States	Non-HHVBP States
**Home Health Utilization (county-year level)**		
FFS beneficiaries with at least 1 HH episode	4,368	20,722
**Health Care Utilization During HH Episodes (claims-based episode level)**		
Unplanned hospitalizations among 1st HH episodes	5,422,915	15,580,576
Total ED use	5,459,773	15,691,141
Outpatient ED use	5,422,915	15,580,576
ED use followed by inpatient admission	5,459,773	15,691,141
SNF use	9,990,283	33,769,633
**Average Medicare Parts A and B Payments Per Day (episode level)**		
During and following FFS HH episodes of care	21,424,943	72,216,849
**Components of Average Medicare Payments Per Day During and Following FFS HH Episodes of Care (claims-based episode level)**		
HH	21,424,943	72,216,849
Inpatient		
Outpatient institutional		
ED and observation stays		
Other		
SNF		
Hospice		
Part B non-institutional [b]		
**OASIS-based Quality Measures (OASIS episode level)**		
Discharged to community	12,416,913	41,153,728
TNC change in self-care	9,258,578	30,008,484
TNC change in mobility	9,258,578	30,008,484
Improvement in dyspnea	7,182,872	22,554,613
Improvement in management of oral medications	7,778,353	24,885,978
Improvement in pain interfering with activity	7,773,002	25,361,828

© 2022 Pozniak A et al. *JAMA Health Forum.*

Measure	HHVBP States	Non-HHVBP States
**Patient Experience (agency-year level)**		
How often the home health team gave care in a professional way (professional care)	12,127	50,021
How well did the home health team communicate with patients (communication)		
Did the home health team discuss medicines, pain, and home safety with patients (discussion of care)		
How do patients rate the overall care from the home health agency (overall care)		
Would patients recommend the home health agency to friends and family (likely to recommend)		

Abbreviations: ED, emergency department; FFS, fee-for-service; HH, home health; HHCAHPS, Home Health Care Consumer Assessment of Healthcare Providers and Systems; HHVBP, home health value-based purchasing; OASIS, Outcome and Assessment Information Set; SNF, skilled nursing facility; TNC, total normalized composite.

© 2022 Pozniak A et al. *JAMA Health Forum.*

eFigure 1. Percentage of Home Health Episodes with an Initial COVID-19 Diagnosis, January to December 2020

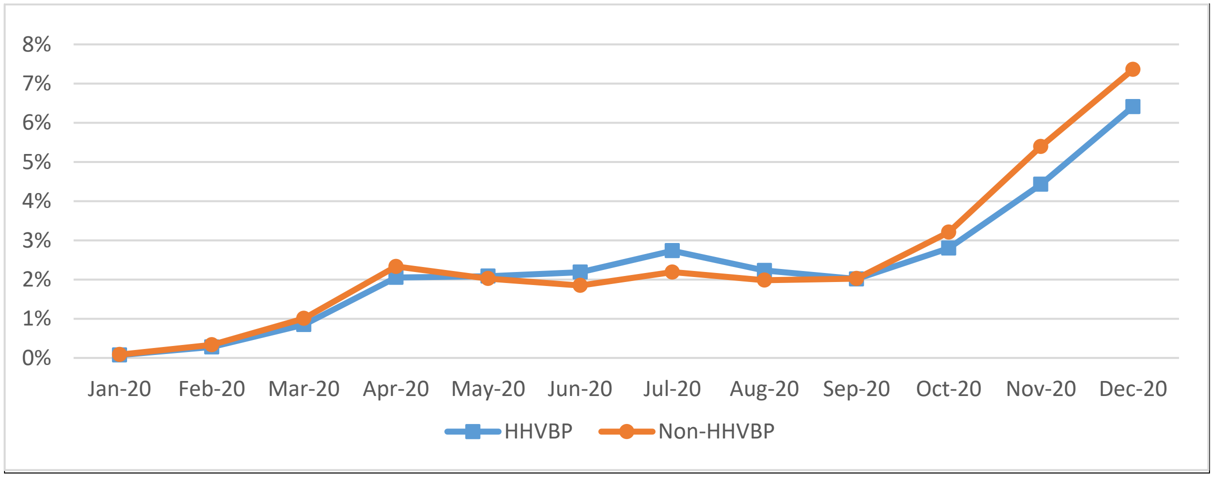

© 2022 Pozniak A et al. *JAMA Health Forum.*

eFigure 2. Assessing Parallel Trends for Outcomes based on Adjusted Models

Legend: Each panel shows the adjusted measure value for HHVBP and non-HHVBP states for 2013-2020

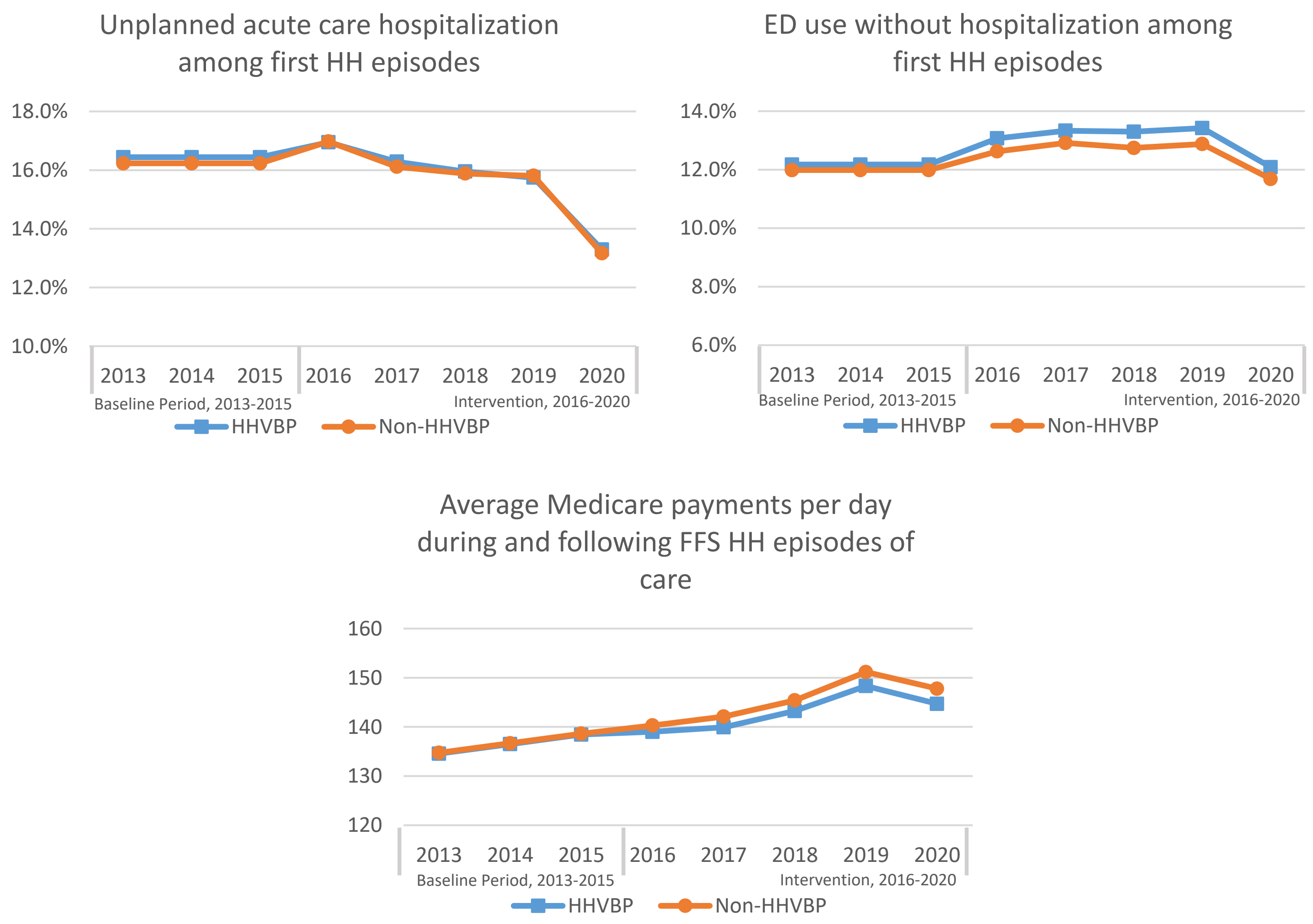

© 2022 Pozniak A et al. *JAMA Health Forum.*

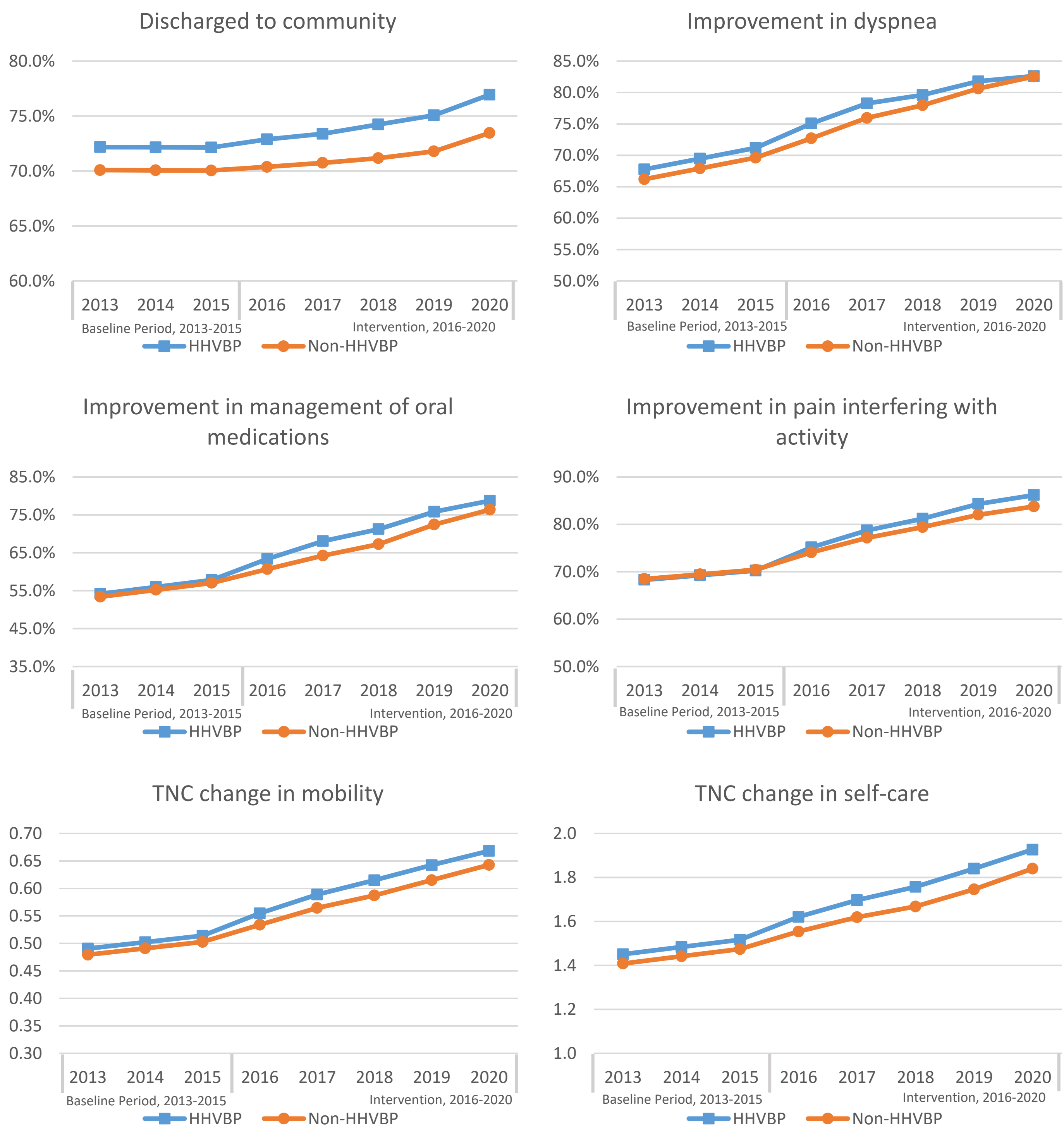

© 2022 Pozniak A et al. *JAMA Health Forum.*

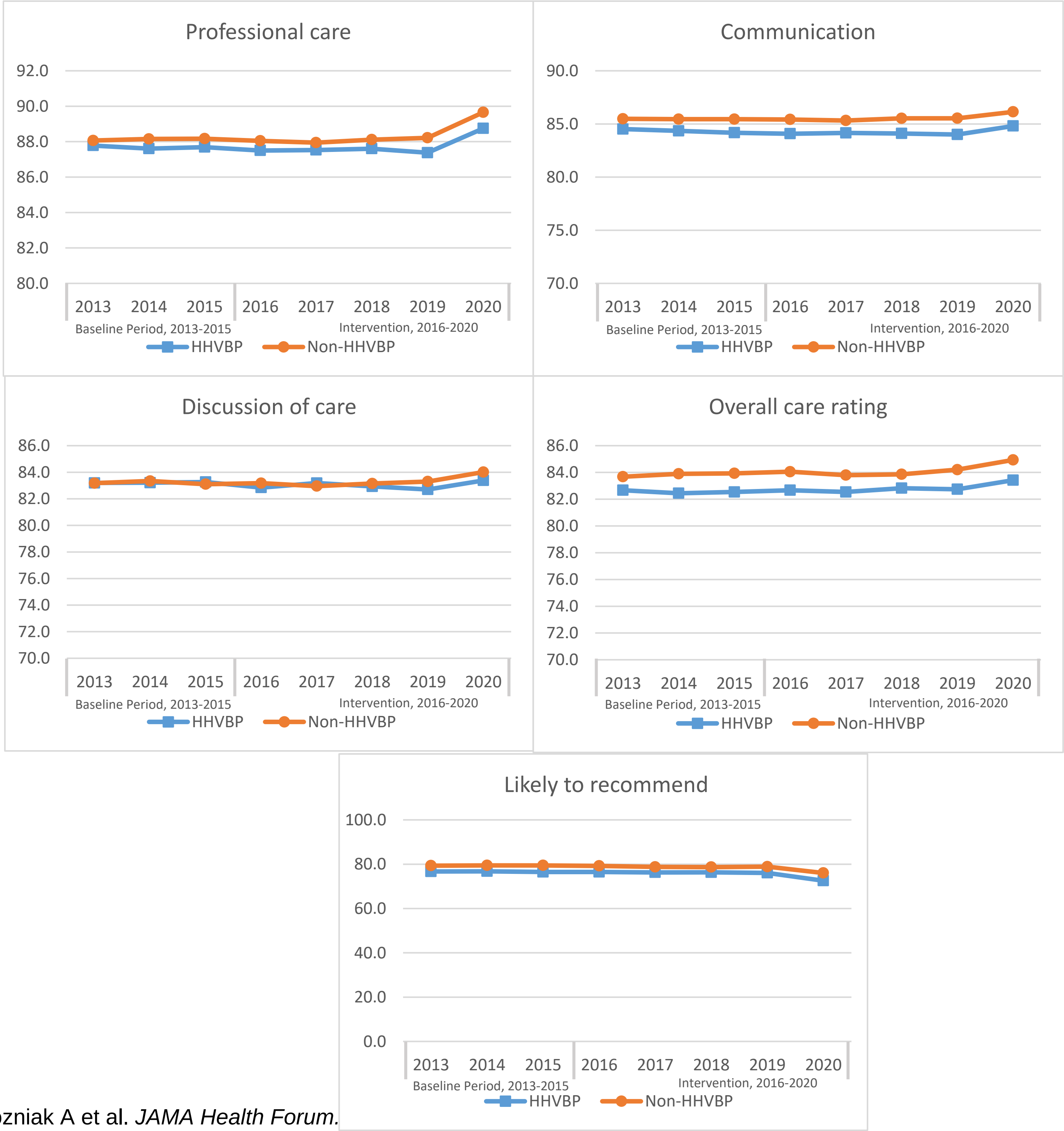

© 2022 Pozniak A et al. *JAMA Health Forum.*
